# Transcriptional mediators of treatment resistance in lethal prostate cancer

**DOI:** 10.1038/s41591-021-01244-6

**Published:** 2021-03-04

**Authors:** Meng Xiao He, Michael S. Cuoco, Jett Crowdis, Alice Bosma-Moody, Zhenwei Zhang, Kevin Bi, Abhay Kanodia, Mei-Ju Su, Sheng-Yu Ku, Maria Mica Garcia, Amalia R. Sweet, Christopher Rodman, Laura DelloStritto, Rebecca Silver, John Steinharter, Parin Shah, Benjamin Izar, Nathan C. Walk, Kelly P. Burke, Ziad Bakouny, Alok K. Tewari, David Liu, Sabrina Y. Camp, Natalie I. Vokes, Keyan Salari, Jihye Park, Sébastien Vigneau, Lawrence Fong, Joshua W. Russo, Xin Yuan, Steven P. Balk, Himisha Beltran, Orit Rozenblatt-Rosen, Aviv Regev, Asaf Rotem, Mary-Ellen Taplin, Eliezer M. Van Allen

**Affiliations:** 1 Harvard Graduate Program in Biophysics, Boston, MA USA; 2 Department of Medical Oncology, Dana-Farber Cancer Institute, Boston, MA USA; 3 Broad Institute of Harvard and MIT, Cambridge, MA USA; 4 Harvard Medical School, Boston, MA USA; 5 Department of Medicine, Division of Hematology/Oncology, Beth Israel Deaconess Medical Center, Boston, MA USA; 6 Center for Cancer Genomics, Dana-Farber Cancer Institute, Boston, MA USA; 7 Columbia Center for Translational Immunology, New York, NY USA; 8 Department of Medicine, Division of Hematology/Oncology, Columbia University Medical Center, New York, NY USA; 9 Department of Immunology, Blavatnik Institute, Harvard Medical School, Boston, MA USA; 10 Evergrande Center for Immunologic Diseases, Harvard Medical School and Brigham and Women’s Hospital, Boston, MA USA; 11 Department of Urology, Massachusetts General Hospital, Boston, MA USA; 12 Division of Hematology and Oncology, University of California, San Francisco, San Francisco, CA USA; 13 Department of Biology, Howard Hughes Medical Institute and Koch Institute for Integrative Cancer Research, MIT, Cambridge, MA USA; 14 Present Address: Department of Pathology, University of Massachusetts Memorial Medical Center, Worcester, MA USA; 15 Present Address: Department of Thoracic/Head and Neck Oncology, MD Anderson Cancer Center, Houston, TX USA; 16 Present Address: Department of Genomic Medicine, MD Anderson Cancer Center, Houston, TX USA; 17 Present Address: Genentech, South San Francisco, CA USA; 18 Present Address: AstraZeneca, Waltham, MA USA

**Keywords:** Prostate cancer, Cancer genomics, Tumour immunology, RNA splicing, Computational biology and bioinformatics

## Abstract

Metastatic castration-resistant prostate cancer is typically lethal, exhibiting intrinsic or acquired resistance to second-generation androgen-targeting therapies and minimal response to immune checkpoint inhibitors^1^. Cellular programs driving resistance in both cancer and immune cells remain poorly understood. We present single-cell transcriptomes from 14 patients with advanced prostate cancer, spanning all common metastatic sites. Irrespective of treatment exposure, adenocarcinoma cells pervasively coexpressed multiple androgen receptor isoforms, including truncated isoforms hypothesized to mediate resistance to androgen-targeting therapies^2,3^. Resistance to enzalutamide was associated with cancer cell–intrinsic epithelial–mesenchymal transition and transforming growth factor-β signaling. Small cell carcinoma cells exhibited divergent expression programs driven by transcriptional regulators promoting lineage plasticity and HOXB5, HOXB6 and NR1D2 (refs. ^4–6^). Additionally, a subset of patients had high expression of dysfunction markers on cytotoxic CD8^+^ T cells undergoing clonal expansion following enzalutamide treatment. Collectively, the transcriptional characterization of cancer and immune cells from human metastatic castration-resistant prostate cancer provides a basis for the development of therapeutic approaches complementing androgen signaling inhibition.

## Abstract (web summary)

Single-cell transcriptomic analysis of metastatic castration-resistant prostate cancer uncovers pervasive coexpression of androgen receptor isoforms and cancer cell–intrinsic and microenvironmental programs of treatment resistance

## Main

Prostate cancer cells depend on the activity of the transcription factor androgen receptor (AR), which represents a major therapeutic target both in primary and advanced disease. Metastatic castration-resistant prostate cancer (mCRPC) is treated with second-generation androgen-targeting therapies, including enzalutamide and abiraterone^1^. Development of resistance to these therapies is nearly universal and neuroendocrine prostate cancer (NEPC) is often intrinsically resistant^1,7^. Despite successes in other solid tumors, immune checkpoint inhibitors have provided minimal benefit except in rare subsets of patients^1^. Both cancer cell–intrinsic and extrinsic programs contributing to resistance remain areas of active investigation. Large-scale genomic analyses have associated alterations in *RB1*, *AR*, *TP53*, Wnt/β-catenin pathway, PI3K pathway and homologous recombination repair genes with worse outcomes on second-generation androgen-targeting therapies^8–11^. Moreover, substantial attention has been focused on a diverse set of AR isoforms, particularly those lacking a ligand-binding domain and hypothesized to be constitutively active^2,3^. As bulk sequencing of biopsies of varying tumor purity collected from diverse metastatic sites, which harbor different nonmalignant cells, may obscure cell type–specific expression programs, we profiled human mCRPC using single-cell transcriptomics.

**Extended Data Fig. 1:**
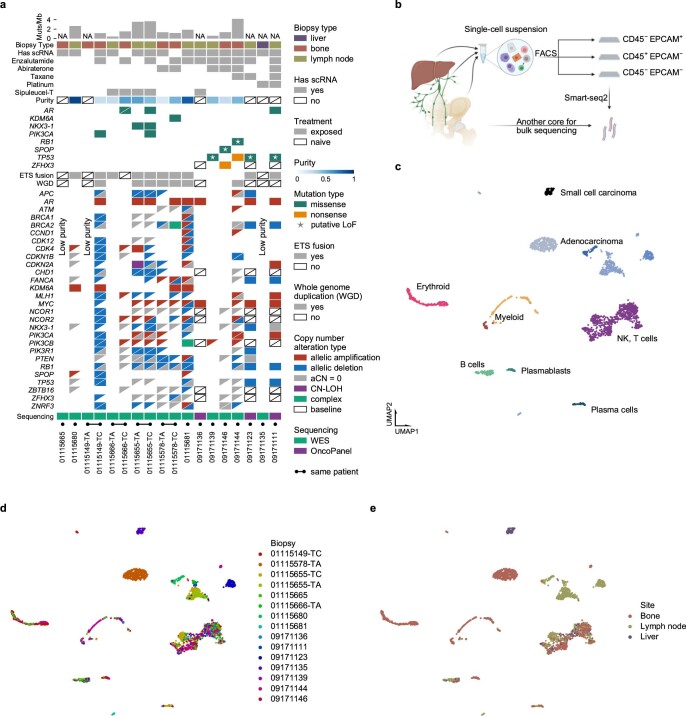
Cellular atlas of mCRPC. **a**, Summary of clinical and select genomics features of patients and biopsies forming the study cohort. Each column represents a single biopsy. Where available, multiple biopsies from the same patient are displayed in adjacent columns. Patients are identified by numerical prefix, while suffixes after a dash, when present, identify biopsies from the same patient. Copy number calls based on whole exome sequencing (WES), but not OncoPanel, are allelic, with calls for the two alleles indicated by two triangles. In cases with whole genome doubling, it is possible for one allele to be amplified and one or both copies of the other allele to be lost. *AR* and *KDM6A* are on the X chromosome and so have only a single copy in these patients; they are represented with solid boxes for copy number status. Boxes with diagonal slashes indicate missing data, for example for genes not included in OncoPanel or for low tumor purity samples for which FACETS does not produce a purity estimate. Putative loss of function (LoF) missense mutations were annotated as LoF or likely LoF in OncoKB or mutated the same amino acid as a LoF mutation^60^. **b**, Study design overview. Dissociated single cells were sorted to enrich for tumor (CD45^-^ EPCAM^+^), immune (CD45^+^ EPCAM^-^), or other populations (CD45^-^ EPCAM^-^). **c**, Projection of single-cell expression onto the first two dimensions of UMAP space. Each dot represents a single cell, and colors correspond to clusters identified by the Louvain algorithm. Clusters are manually labelled with dominant cell type(s) inferred from cluster-specific expression of marker genes. Cells colored corresponding to (**d**) biopsy of origin or (**e**) metastatic site. Non-malignant cells from different patients jointly cluster by cell type, while cancer cells from different patients largely form distinct clusters.

**Extended Data Fig. 2:**
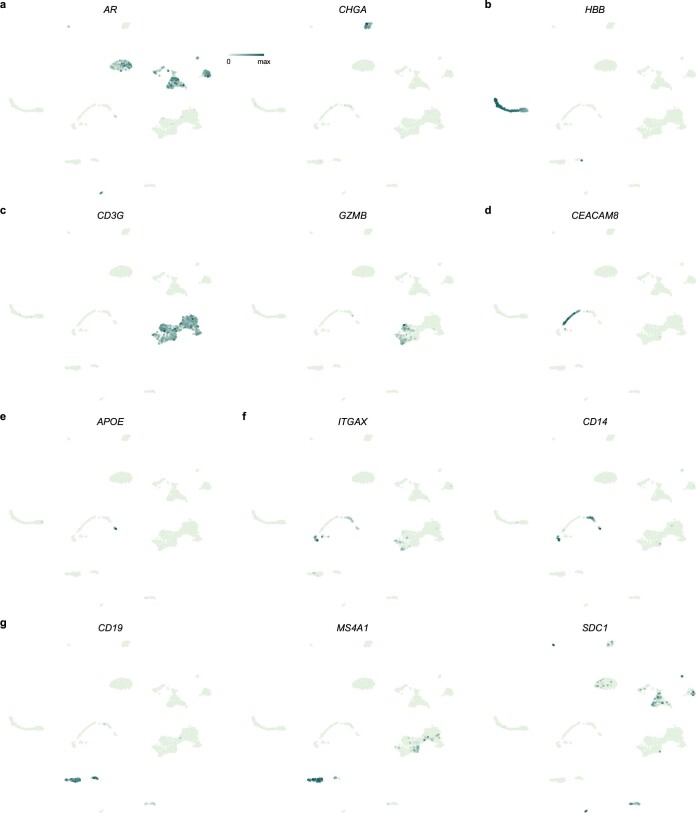
Marker gene expression used for cluster labeling. Expression of select cell type marker genes for (**a**) prostate cancer cells (*AR* is expressed in adenocarcinoma, and *CHGA* is expressed in small cell carcinoma) (**b**) erythroid cells (**c**) T and NK cells (see also Extended Data Fig. 4) (**d**) neutrophils (**e**) macrophages (**f**) monocyte subsets, and (**g**) B lineage cells (see Methods for details on using combinations of markers to distinguish B cells, plasmablasts, and plasma cells). UMAP projections as in Extended Data Fig. 1c.

With informed consent, we collected fresh biopsies from the three common mCRPC metastatic sites (bone, lymph node and liver) for whole exome sequencing, bulk RNA sequencing (RNA-seq) and single-cell RNA-seq (scRNA-seq) using the Smart-seq2 protocol (Methods)^12^. Patients had varied treatment histories, with biopsies approximately evenly represented before and after enzalutamide exposure (Extended Data Fig. 1 and Supplementary Table 1). In addition to adenocarcinomas, one tumor exhibited a small cell carcinoma histology. After quality control, our cohort consisted of 2,170 cells from 14 patients and 15 biopsies (Methods; Supplementary Table 3). We labeled clusters for dominant cell type based on marker gene expression (Extended Data Fig. 2; Methods).

**Fig. 1:**
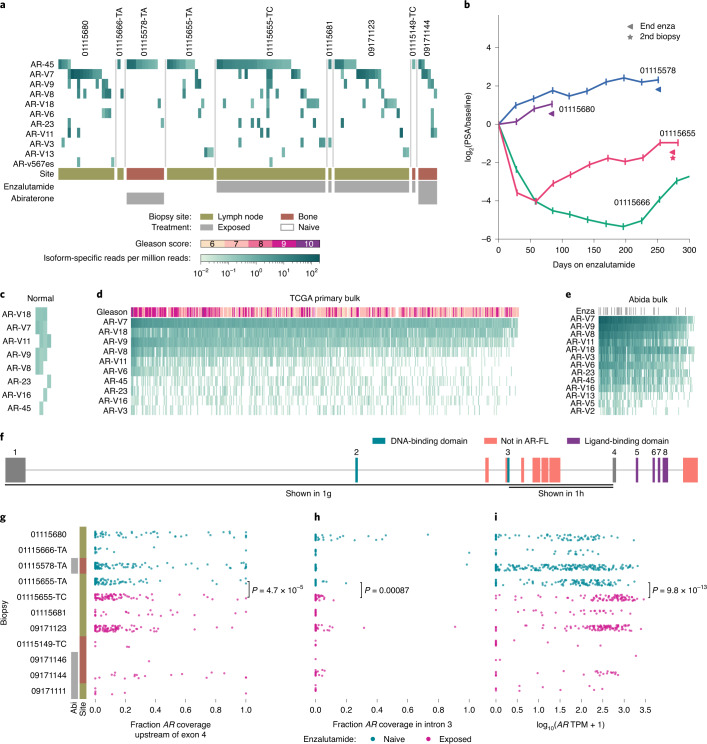
Complex AR isoform coexpression within individual cells and tumors is common across disease stages and resistance states. **a**, Heat map displaying frequency of isoform-specific reads mapping to AR splice variants (Supplementary Fig. 1). Each column represents AR variants detected in a single cell, with only cells and isoforms that had at least one isoform-specific read shown. Short-read-based approaches cannot specifically identify full-length AR (Methods). **b**, PSA histories for the four patients for whom immediately pre-enzalutamide biopsies are shown in **a**. Patient 01115666 remained on enzalutamide beyond the 300 d shown. **c**–**e**, Heat maps displaying frequency of isoform-specific reads in bulk RNA-seq of healthy prostate (*n* = 4)^15^, TCGA primary prostate adenocarcinoma (*n* = 496)^14^ and advanced prostate adenocarcinoma (*n* = 127, poly-A-sequenced only, enzalutamide and abiraterone-naive or enzalutamide-exposed)^8^. Gleason score and enzalutamide exposure status are shown for TCGA and Abida et al. cohorts, respectively. For each cohort, AR variants detected in at least 20% of samples are shown. **f**, Schematic representation of *AR* locus. Rectangles indicate exons. Exons corresponding to the full-length *AR* transcript are numbered, with exons comprising different functional domains colored. Select alternative exons included in AR splice variants are indicated. **g**, Fraction of total *AR* coverage upstream of exon 4 (including the DNA-binding domain but excluding the ligand-binding domain) in single cells. **h**, Fraction of total *AR* coverage in intron 3 (including multiple cryptic/alternative exons included in truncated splice variants) in single cells. **i**, Total *AR* expression in single cells. **g**–**i***, P* value compares cells before (*n* = 112) and after (*n* = 83) enzalutamide treatment for patient 01115655 (two-sided Mann–Whitney *U-*test).

To investigate the role of AR isoforms in resistance, we developed methodology to specifically identify literature-described isoforms (Supplementary Figs. 1 and 2a,b; Methods). Isoform-specific reads corresponding to multiple AR variants were detected in the same cell across tumors and treatment exposures. AR-V7, AR-V8 and AR-V9, which lack the ligand-binding domain, were present both in cells exposed to and naive to second-generation androgen-targeting compounds (Fig. 1a). All three were detected in enzalutamide- and abiraterone-naive cells from patient 01115655, who nonetheless experienced a large decrease in prostate-specific antigen (PSA) levels following enzalutamide initiation (Fig. 1b). In post-enzalutamide cells from the same patient, we observed no evidence of a selective sweep driven by any dominant single AR isoform, and almost all post-treatment isoforms were detectable in some cells before enzalutamide. In cancer cells from patient 01115578, who had no clinical response to enzalutamide, we did not robustly detect any isoform, except AR-45 (hypothesized to be dominant negative)^2,13^. Across the cohort, AR-45 and AR-V7 were coexpressed in significantly fewer cells than expected by chance, but we did not observe a replacement of AR-45-expressing cells by AR-V7-expressing cells in post-enzalutamide biopsies (Supplementary Fig. 2c). More generally, we did not consistently detect any isoform specific to post-enzalutamide biopsies.

**Extended Data Fig. 3:**
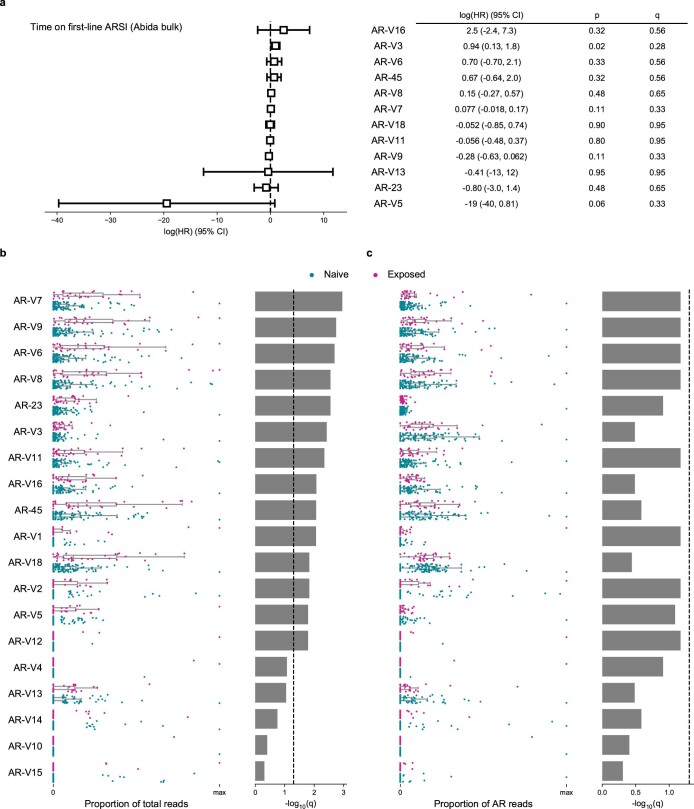
Association of AR variant expression with clinical features in advanced prostate cancer. Analysis of isoform-specific read frequency in poly-A-sequenced samples with an adenocarcinoma histology from Abida et al. cohort^8^. **a**, Summary of multivariate Cox proportional hazards analysis of time on first-line androgen receptor signaling inhibitor (ARSI; abiraterone or enzalutamide) using AR variant frequency (isoform-specific reads / million total reads) in pre-treatment ARSI-naïve samples (n = 46). AR variants detected in at least 20% of samples were included. 95% confidence intervals and *p-* values are unadjusted for multiple hypotheses. *q* values correspond to Benjamini-Hochberg FDR. Comparison of expression levels of AR variants measured as a proportion of (**b**) all reads or (**c**) *AR* reads in ARSI-naïve (n = 98) vs. enzalutamide-exposed patients (n = 29). In stripplots, each dot corresponds to a single tumor. Bar plots show *q* values from Benjamini-Hochberg adjustment of *p* values from two-sided Mann Whitney *U* tests. Dashed lines indicate *q* = 0.05. Boxplots: center line: median; box limits: upper and lower quartiles; whiskers extend at most 1.5x interquartile range past upper and lower quartiles.

Applying our approach to bulk RNA-seq of normal prostate, TCGA primary prostate adenocarcinoma and mCRPC adenocarcinomas, we consistently detected coexpression of multiple isoforms within the same tumor across disease stages (Fig. 1c–e)^8,14,15^. AR-V7, AR-V8 and AR-V9 were detected even in normal prostate and in mCRPC, no AR isoform was significantly associated with duration on treatment (Extended Data Fig. 3a). While we observed robust increases in AR-V7 after enzalutamide exposure, increases were observed for most of the detected isoforms (Extended Data Fig. 3b). After normalizing the number of isoform-specific reads to *AR* read count (as opposed to total read count), we observed no significant differences between exposed and naive tumors (Extended Data Fig. 3c). These observations suggest that the increased abundance after enzalutamide exposure of many isoforms (including AR-V7) may largely be a consequence of increased total *AR* expression and that the relative abundances of the different isoforms may be largely unchanged. Similarly, higher Gleason scores were more common in primary tumors with increased abundance of many AR isoforms but not of a specific variant (Fig. 1d). Collectively, these results suggest no single AR isoform is a major driver of resistance to therapy.

Substantial numbers of reads from the *AR* genomic interval failed to map to any curated isoform, suggesting described isoforms form an incomplete census (Supplementary Fig. 2d). Short-read sequencing precludes direct isoform reconstruction, so we defined two measures of *AR* splicing that may be functionally informative. Intron 3 contains many of the terminal cryptic/alternative exons included in truncated isoforms, so we quantified the proportion of total *AR* coverage from intron 3 or from a larger interval that includes upstream exons (Fig. 1f)^2^. Increases in these measures may suggest expression of a greater proportion of transcripts that encode ligand-independent proteins.

We detected an increase in both measures after enzalutamide treatment for patient 01115655, but the more dramatic effect was an increase in total *AR* expression and copy number (Fig. 1g–i). Across samples, wide variation in cells from the same tumor was pervasive. Some enzalutamide-exposed biopsies (09171123 and 0917144) had high proportions of cells with high values for both measures, but so did some number of cells from all enzalutamide-naive biopsies, including from both patients with subsequent PSA response. More generally, the observed isoform coexpression patterns muddy interpretation of their functional effects, especially given poorly understood heterodimerization^2,13^. Combined with the lack of systematic differences before and after therapy, our observations suggest that assessments of single AR variants may be insufficient for a causal understanding of a tumor’s sensitivity to androgen-targeting therapies even if their detection can serve as a proxy of total AR.

**Fig. 2:**
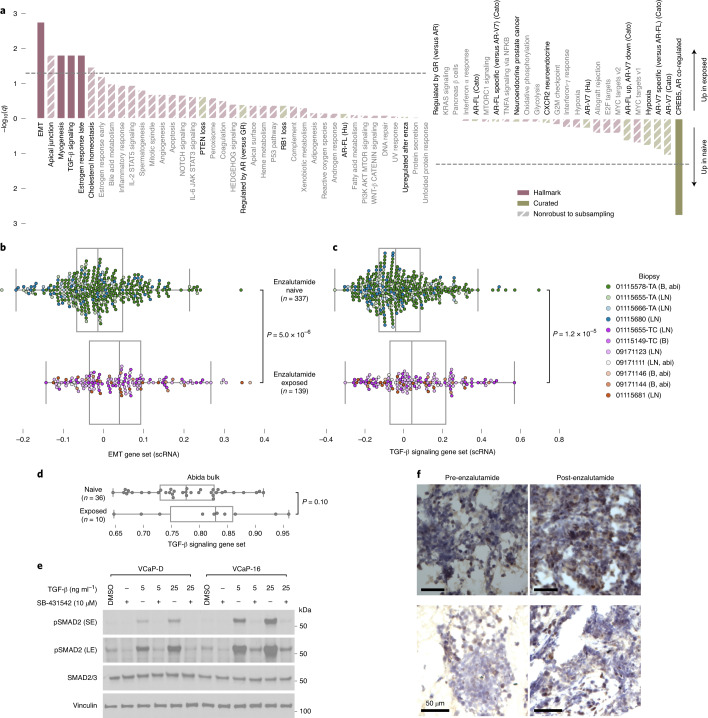
Enzalutamide-exposed adenocarcinoma cells upregulate expression programs associated with EMT and TGF-β signaling. **a**, Comparison of gene set expression scores in enzalutamide-exposed versus naive adenocarcinoma cells. Gene sets included include the MSigDB hallmark collection^16^ and literature-curated gene sets hypothesized to be related to enzalutamide resistance (Supplementary Table 5). *q* values from Benjamini–Hochberg adjustment of *P* values from two-sided Mann–Whitney *U*-tests (Supplementary Table 6). The broken lines indicate *q* = 0.05. Only G1 cells were included in analyses. Results shown are the median of a subsampling procedure designed to even out representation of cells from different biopsies. Each subsample included 67 exposed and 76 naive cells. Hashed bars correspond to results that were nonrobust in an additional leave-one-sample-out sampling step, suggesting that the effect is driven by patient-specific mechanisms. For details of subsampling used during statistical testing, see Methods and Supplementary Fig. 3. enza, enzalutamide; IL, interleukin; GR, glucocorticoid receptor. **b**,**c**, MSigDB hallmark EMT (**b**) and TGF-β signaling gene set expression scores (**c**) for individual cells (G1 only) collected before and after enzalutamide treatment. Each dot represents a single cell and is colored corresponding to biopsy. Biopsy clinical attributes are indicated in parentheses in legend (B, bone biopsy; LN, lymph node biopsy; abi, previous abiraterone exposure). *P* values from two-sided Mann–Whitney *U*-test, including all G1 cells. **d**, MSigDB hallmark TGF-β signaling gene set expression scores for bulk RNA-seq of mCRPC lymph node biopsies^8^ collected before and after enzalutamide treatment. Each dot represents a single tumor. *P* value from one-sided Mann–Whitney *U*-test. **e**, Western blot of SMAD2/3 and phospho-SMAD2 levels in enzalutamide-sensitive VCaP-D and enzalutamide-resistant VCaP-16. Cells were grown in basal maintenance medium with 0.5% FBS for 48 h, then treated for 2 h with the TGF-β receptor inhibitor SB-431542, recombinant TGFβ-1 protein or a combination of both. Cells treated with SB-431542 were pretreated for 24 h with SB-431542 before addition of recombinant TGFβ-1 protein. SE, short exposure; LE, long exposure. For uncropped images and both sets of vinculin loading controls, see Source Data. DMSO, dimethylsulfoxide. **f**, Immunohistochemical staining of phospho-SMAD2 in longitudinal biopsies from two patients immediately before and after enzalutamide treatment. Each row corresponds to one patient. All biopsies are from bone metastases. Scale bars, 50 μm. Box plots are represented by center line, median; box limits, upper and lower quartiles; whiskers extend at most 1.5× interquartile range past upper and lower quartiles. Source data

Next, we sought to identify broad expression changes following enzalutamide exposure. We scored cancer cells for expression of the MSigDB hallmark gene sets and curated literature-derived gene sets corresponding to proposed resistance mechanisms using a subsampling procedure to filter out patterns driven by individual tumors (Fig. 2a; Methods)^16–27^. Enzalutamide-exposed cells upregulated gene sets for epithelial–mesenchymal transition (EMT) and transforming growth factor (TGF)-β signaling (Fig. 2a–c). We observed consistent increases when restricting only to the patient with paired biopsies (Supplementary Fig. 4a,b). In a cohort of mCRPC bulk RNA-seq, we found a similar effect for TGF-β signaling in enzalutamide-exposed lymph node biopsies, although the number of exposed biopsies was small and the effect was not statistically significant (Fig. 2d)^8^. EMT scores were confounded with tumor purity in these bulk data, with large differences likely driven by presence of benign cells (Supplementary Fig. 4c)^28^.

Concordantly, in VCaP-16, a cell line generated from long-term culture in enzalutamide until emergence of resistance, we observed increased SMAD2 phosphorylation following equivalent TGF-β stimulation compared to enzalutamide-sensitive VCaP-D cells, indicating they are primed to amplify downstream signaling by TGF-β (Fig. 2e; Methods). Short-term exposure of wild-type VCaP cells to enzalutamide had minimal effect (Supplementary Fig. 4d). Furthermore, in two pairs of patient biopsies collected immediately before enzalutamide initiation and after progression, nuclear localized phosphorylated SMAD2 increased in the post-treatment biopsies (Fig. 2f). While other hypothesized resistance programs were not robustly upregulated in our cohort, our observations support clinical studies of TGF-β inhibition in the context of enzalutamide resistance (Fig. 2a).

**Fig. 3:**
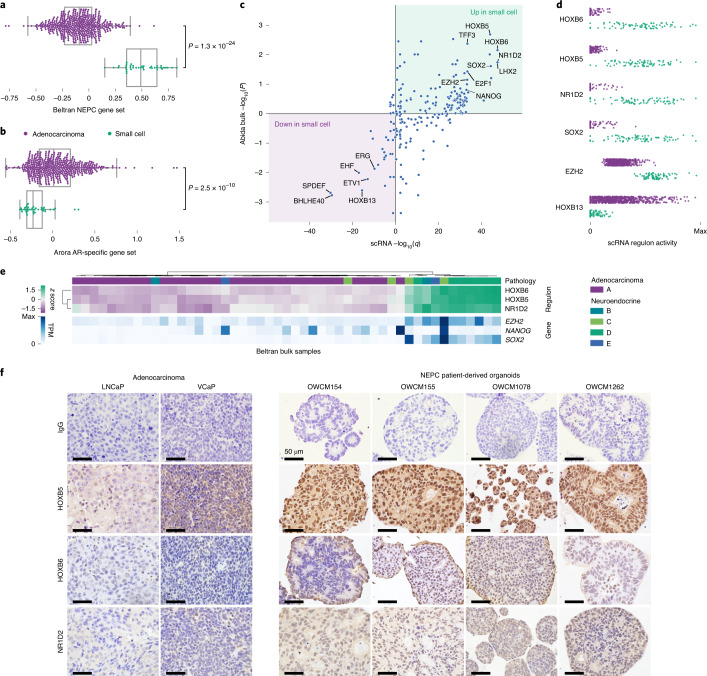
Cancer cells from small cell carcinoma employ a highly divergent regulatory program compared to adenocarcinoma cells. **a**,**b**, Gene set expression scores of single G1 cells using an expression signature of NEPC^18^ (**a**) and a set of genes under regulation by AR^17^ (**b**). Box plots include center line, median; box limits, upper and lower quartiles; whiskers extend at most 1.5× interquartile range past upper and lower quartiles; *P* values are from two-sided Mann–Whitney *U*-test. **c**, Inferred activity of regulons of different transcriptional regulators. *x* axis, *q* values from comparison of inferred regulon activity in cancer cells from small cell carcinoma (*n* = 76) versus cancer cells from adenocarcinomas (*n* = 188, sampled as described in Methods) (negative values indicate regulon is less active in small cell carcinoma; two-sided Mann–Whitney *U*-test, median outcome of sampling iterations (Methods) with Bonferroni FWER correction). *y* axis: *P* values (two-sided Mann–Whitney *U*-test, signed as previous) from comparison of expression scores of scRNA-derived regulons in bulk RNA-seq of small cell carcinomas (*n* = 8) versus adenocarcinomas (*n* = 18) from a published cohort^8^. **d**, Regulon activity in single cells for select transcriptional regulators. **e**, Hierarchical clustering of bulk RNA-seq of a published cohort of prostate cancers of known histopathology^18^ based on expression of HOXB5, HOXB6 and NR1D2 regulons inferred from scRNA-seq. B–E correspond to different NEPC subtype labels from original publication. Expression levels of *EZH2*, *NANOG* and *SOX2* are shown for reference but were not used in clustering (*n* = 34 adenocarcinoma, 15 NEPC). **f**, Immunohistochemical staining of HOXB5, HOXB6 and NR1D2 protein levels in two prostate adenocarcinoma xenografts (LNCaP and VCaP) and four NEPC patient-derived organoids. Scale bar, 50 μm.

One biopsy was of a small cell carcinoma, a rare, aggressive form of prostate cancer that does not respond to androgen-targeting therapies^7^. Cancer cells from this biopsy had no detectable *AR*, downregulated an AR-regulated gene set and upregulated a NEPC gene set (Fig. 3a,b and Supplementary Fig. 5)^17,18^. With only one NEPC biopsy, we analyzed expression levels of gene sets inferred in our scRNA-seq to be regulated by specific transcription factors (regulons), an approach shown to identify more generalizable expression programs (Methods)^29^.

Comparing the single-cell results and a bulk-sequenced cohort that we scored for expression of the regulons inferred in scRNA-seq, we observed concordant patterns of differential regulon activity between adenocarcinoma and small cell carcinoma (Fig. 3c; Methods)^8^. Adenocarcinomas exhibited higher activity of HOXB13, which mediates AR regulatory activity, and of several ETS family transcription factors^30^. Small cell carcinoma had higher activity of NANOG, SOX2 and EZH2, which promote lineage plasticity and resistance to androgen-targeting therapies^4–7^. Additionally, we observed increased activity of three transcriptional regulators whose role in NEPC has not been reported: HOXB5 and HOXB6, two homeobox containing transcription factors and NR1D2, a nuclear hormone receptor family member (Fig. 3c,d). In a second, independent, bulk-sequenced clinical cohort, inferred regulon activity of these transcription factors partitioned tumors by histopathology classification (Fig. 3e)^18^. Furthermore, all three were consistently expressed in NEPC patient-derived organoids but not in adenocarcinoma models (Fig. 3f). In aggregate, we recovered generalizable patterns of cancer cell–intrinsic expression in NEPC, recovering known regulators mediating resistance and implicating new transcription factors.

**Fig. 4:**
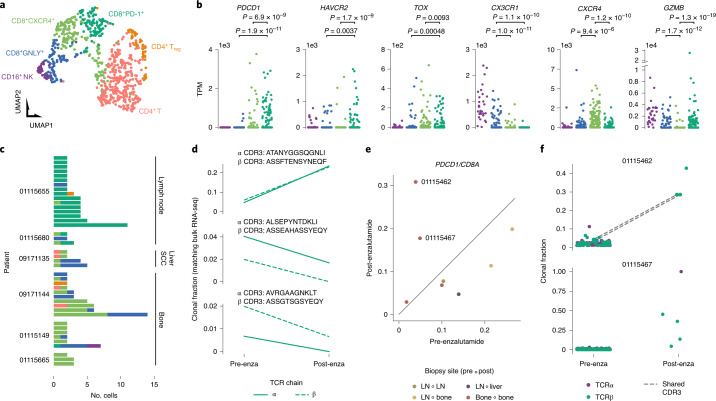
Cytotoxic lymphocyte populations and clonal expansions in metastatic niches. **a**, Subclustering of NK and T cells. Each dot represents a single cell projected onto uniform manifold approximation and projection (UMAP) space colored corresponding to clustering via the Louvain algorithm. Clusters are manually labeled with dominant phenotype/cell type from patterns of marker gene expression. Cluster colors are used throughout subpanels. **b**, Expression of select marker, effector and co-inhibitory receptor genes within cytotoxic clusters, CD16^+^ NK (*n* = 30), CD8^+^GNLY^+^ (*n* = 84), CD8^+^CXCR4^+^ (*n* = 157) and CD8^+^PD-1^+^ (*n* = 106). Nominal *P* values from a two-sided Mann–Whitney *U*-test are shown. TPM, transcripts per million. **c**, T cell clonotypes from scRNA-seq TCR reconstruction. Each bar represents cells sharing a reconstructed productive TCR CDR3 sequence. Bars are grouped by patient. SCC, small cell carcinoma. **d**, Changes in clonal fractions of cytotoxic T cell clonotypes in patient 01115655 following enzalutamide treatment. Each subplot corresponds to a single clonotype with TCRα and β CDR3 amino acid sequences paired from scRNA-seq. Clonal fractions for the same CDR3 sequences (matching at both nucleotide and amino acid level) inferred from TCR reconstruction in bulk RNA-seq are plotted. All detected single cells of the displayed clonotypes came from the CD8^+^PD-1^+^ T cell cluster. **e**, *PDCD1* to *CD8A* expression ratio in bulk RNA-seq of paired biopsies from the same patient before and after enzalutamide treatment. Paired biopsies did not always derive from the same site; color of dots indicate biopsy site types. For patient 01115462, the pre-enzalutamide biopsy was collected from the sacrum and the post-enzalutamide biopsy was collected from a left ischial lesion. **f**, Expanded T cell clonotypes following enzalutamide in patients 01115462 and 01115467. Each dot represents a single CDR3 sequence detected in bulk RNA-seq. CDR3 sequences identified both before and after enzalutamide are connected by a broken gray line.

**Extended Data Fig. 4:**
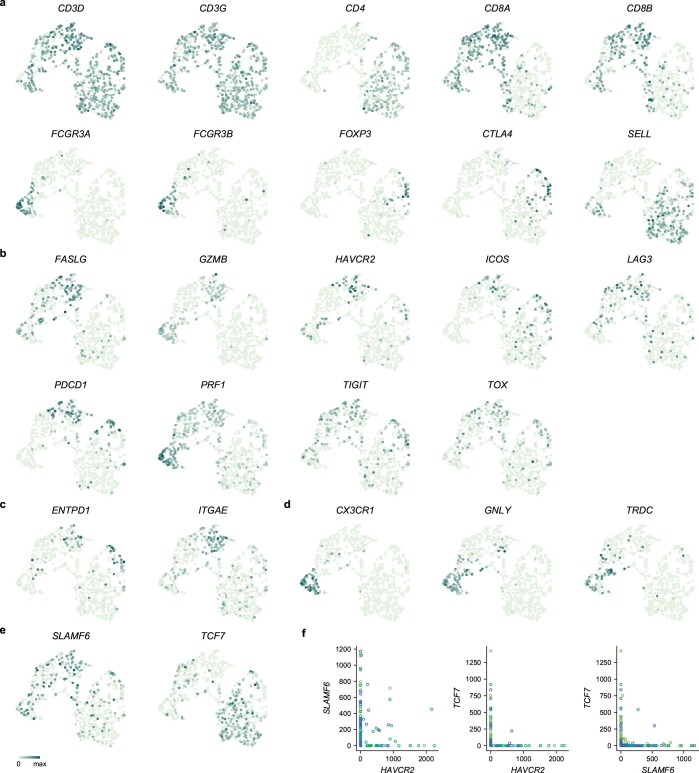
Marker gene expression in NK and T cells. Expression of (**a**) cell type markers, (**b**) dysfunction and activation markers, (**c**) markers of tumor-reactive cytotoxic cells^31^, (**d**) genes expressed in a *GNLY*-expressing cytotoxic subset, and (**e**) genes reported to mark a progenitor population necessary for response after anti-PD-1 therapy in melanoma^32^. Cells are projected onto UMAP space as in Fig. 4a. **f**, Scatterplots showing pairwise co-expression of *HAVCR2*, *SLAMF6*, and *TCF7* in CD8^+^ T cells. Points are colored according to cluster membership as in Fig. 4a.

To complement therapeutic strategies targeting cancer cell–intrinsic mechanisms, clinical trials have tested immune checkpoint inhibitors in prostate cancer, but responses in mCRPC have been limited^1^. We characterized infiltrating cytotoxic cells from various metastatic niches to improve understanding of mCRPC immunobiology. We subclustered T and natural killer (NK) cells into two CD4^+^ T cell populations, three largely CD8^+^ T cell populations and a population of strongly CD16^+^ and largely CD3^−^ cells dominated by NK cells (Fig. 4a and Extended Data Fig. 4a). Aside from a population of *CXCR4*-expressing CD8^+^ T cells chiefly derived from bone biopsies, cytotoxic clusters exhibited widespread *GZMB* expression (Fig. 4b and Supplementary Fig. 6a). One CD8^+^ T cell population largely derived from lymph node biopsies was marked by expression of *PDCD1* (encoding PD-1), *HAVCR2*, *TOX*, *TIGIT*, *ICOS*, *FASLG* and *LAG3*, suggestive of a dysfunctional effector phenotype (Fig. 4b and Extended Data Fig. 4b,c). This population also exhibited elevated expression of both *ENTPD1* and *ITGAE*, whose coexpression identifies infiltrating cytotoxic cells reactive to cancer cells in other human cancers^31^. We did not observe a distinct cluster of *TCF7* and *SLAMF6* dual-expressing progenitor cells reported to mediate response to anti-PD-1 therapy in melanoma, but it remains unclear whether this explains poor response to immune checkpoint inhibitors in mCRPC (Extended Data Fig. 4e,f)^32^.

To identify potential immune system responses to metastasis, we examined T cell clonal expansion by reconstructing T cell receptor (TCR) complementarity-determining region 3 (CDR3) sequences in individual cells and matching bulk RNA-seq (Methods). Groups of T cells forming part of an expanded clonotype group with a shared CDR3 sequence were detected in six patients. Clonotype groups detected in lymph node metastases largely comprised cells from the CD8^+^ T cell cluster with elevated co-inhibitory receptor expression, whereas clonotype groups detected in bone metastases largely comprised cells from the *CXCR4*-expressing CD8^+^ T cell cluster (Fig. 4c). In one enzalutamide-exposed bone biopsy (09171144), a large clonotype group included cells with high *CX3CR1* expression. CD8^+^ T cells with high effector molecule, low exhaustion marker and *CX3CR1* expression have recently been characterized as highly cytolytic but poorly proliferative and unresponsive to PD-L1 blockade^33–35^.

In patient 01115655, we collected cells from before and after enzalutamide treatment and noted marked changes in the infiltrating T cell populations. Before treatment, cytotoxic cells formed a minority of infiltrating T cells, but following treatment, most infiltrating T cells were dysfunctional *PDCD1*-expressing CD8^+^ T cells. For the largest clonotype group in this patient, we detected the corresponding TCRα and TCRβ CDR3 sequences in bulk RNA-seq from both time points. The clonal fraction estimated from bulk RNA-seq increased sharply from approximately 5% before treatment to 25% after treatment, making it the largest detected clone (Fig. 4d). All cells of this clonotype group detected in scRNA-seq were part of the *PDCD1*-expressing dysfunctional cluster. To assess whether this phenomenon occurs in other patients, we examined a longitudinal mCRPC cohort (NCT01942837) with bulk RNA-seq, comparing biopsies collected before and after enzalutamide treatment. As a measure of the abundance of CD8^+^ T cells resembling those clonally expanded in 01115655, we examined the ratio of *PDCD1* to *CD8A* expression (Fig. 4e). Two patients had marked increases in this ratio following enzalutamide treatment and in both, we observed reduced T cell diversity after enzalutamide. Owing to increased tumor purity in enzalutamide-exposed biopsies, we generally detected fewer clonotypes after treatment; however, in patient 01115462, we detected large increases in clonal fraction of two CDR3 sequences (Fig. 4f).

The observed clonal expansions paired with relatively increased *PDCD1* expression suggests that in some tumors, CD8^+^ T cells do mount an antitumor response in the context of enzalutamide exposure. This may explain why some patients respond to immune checkpoint inhibition following enzalutamide resistance^36^. While atezolizumab, an anti-PD-L1 therapy, was recently reported to provide no benefit in conjunction with enzalutamide, the observation of expanded T cell clones after enzalutamide may indicate that tumor killing by enzalutamide is necessary to drive the tumor immunity cycle^37^. At the same time, high *CX3CR1* expression on some expanded clones may suggest poor response in other patients. Functional assays of infiltrating cytotoxic cells in heavily treated mCRPC are needed to decipher the effects of previous therapy and metastatic site on tumor reactivity and potential response to immunomodulatory therapies.

**Extended Data Fig. 5:**
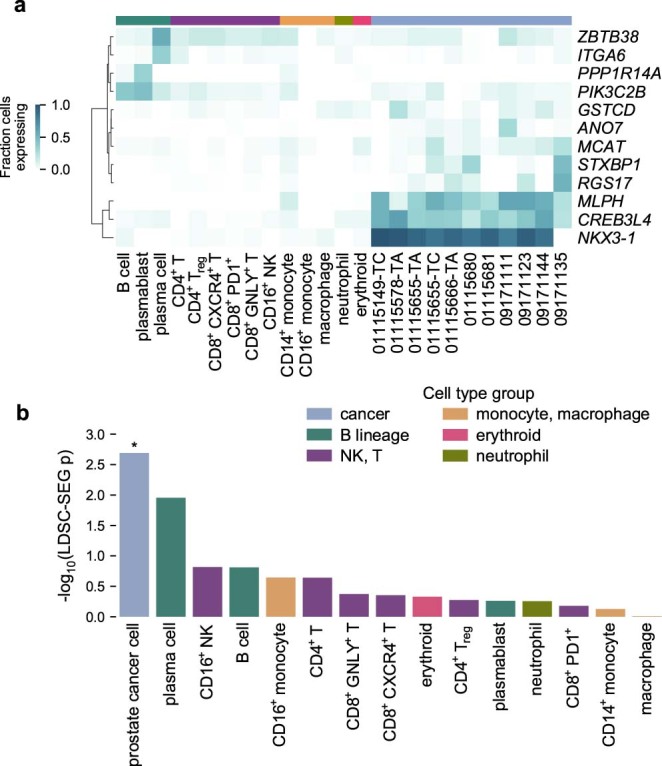
Germline heritability for prostate cancer is enriched near genes specifically expressed in prostate cancer cells. **a**, Expression of a subset of a published set of prostate cancer risk genes that show cell type specificity (Methods)^38^ (**b**) LDSC-SEG enrichment of heritability for prostate cancer near genes specifically expressed in each cell type (compared to cell types in other cell type groups) (Methods). *: Benjamini-Hochberg FDR < 0.05.

To explore the significance of cell populations other than cancer cells and T cells, we applied approaches leveraging genome-wide association studies (GWAS) of inherited prostate cancer risk. We began by examining cell type specificity of 109 genes implicated in an analysis of a GWAS of over 100,000 men (Methods)^38,39^. Genes with cell type-specific expression were dominated by cancer cell-specific genes (Extended Data Fig. 5a). Using linkage disequilibrium score regression applied to specifically expressed genes (LDSC-SEG) to directly integrate GWAS summary statistics with scRNA-seq expression patterns, we likewise found germline heritability enriched only in genomic intervals near genes specifically expressed in cancer cells (Extended Data Fig. 5b; Methods)^40^. These results suggest that any inherited risk for prostate cancer still operant during advanced disease is primarily mediated through cancer cell–intrinsic mechanisms.

In summary, we used single-cell transcriptomics and validation in external cohorts to characterize mCRPC expression programs in the context of therapy. In adenocarcinomas, our findings suggest therapeutic targeting of specific AR isoforms may be insufficient but support clinical inhibition of TGF-β and further characterization of cytotoxic cells. HOXB5, HOXB6 and NR1D2 warrant functional study and potential therapeutic development in NEPC. Longitudinal studies integrating multi-omics and spatially resolved expression in larger cohorts may inform additional mediators of clinical resistance.

## Methods

### Statistical computing

Except where otherwise specified, analysis and data visualization were performed with Python 3.7, SciPy v.1.3.2, Matplotlib v.3.1.1, seaborn v.0.9.0 and R v.3.5.1.

### Reference versions

We used human genome reference b37 and the GENCODE release 30 gene annotation lifted over to GRCh37.

### Whole exome analyses

For biopsies with paired tumor and normal samples available, we performed whole exome sequencing with a customized version of a previously described protocol^41^. After DNA shearing, hybridization and exome capture were performed using Illumina’s Rapid Capture Exome kit (except for the normal sample for 01115149 and the tumor sample for biopsy 01115149-TA, which used the Agilent SureSelect Human All Exon 44 Mb v.2.0 bait set). Libraries were sequenced with 76 bp paired-end reads on an Illumina instrument.

Reads were aligned using BWA v.0.5.9 (ref. ^42^). Somatic mutations were called using a customized version of the Getz Lab CGA WES Characterization pipeline (https://portal.firecloud.org/#methods/getzlab/CGA_WES_Characterization_Pipeline_v0.1_Dec2018/2) developed at the Broad Institute. Copy number alterations, purity, ploidy and whole genome doubling status were called using FACETS v.0.5.14 (ref. ^43^; Supplementary Table 2). Copy number alterations were evaluated with respect to whole genome doubling status. Summary visualization of mutation and copy number status integrated with clinical variables was performed with a beta version of CoMut^44^.

For biopsies where whole exome sequencing failed, somatic mutation calls, copy number alterations and ETS fusion status were taken where available from OncoPanel, a clinical panel sequencing test available at Dana-Farber Cancer Institute.

### Sample collection and dissociation for single-cell RNA-seq

Samples were collected with informed consent and ethics approval by the Dana-Farber/Harvard Cancer Center Institutional Review Board under protocol nos. 09-171, 11-104, 13-301 and 01-045. Tumor samples were collected and transported in Dulbecco’s modified Eagle medium, high glucose, pyruvate (DMEM; Thermo Fisher Scientific, 11995073) on ice. Single-cell suspensions for scRNA-seq were obtained from tumor core needle biopsies through mechanical and enzymatic dissociation. Samples were first cut into pieces <1 mm^3^ using a scalpel. For bone biopsies, soft tissue was also scraped from the hard bone surface using a scalpel blade. Samples were then dissociated using one of two protocols, chiefly to optimize for yield of viable cells from different metastatic sites. Cells obtained from the two protocols were comparable and findings were consistent in subanalyses of cells processed with the same protocol (Supplementary Fig. 7).

For biopsies 01115655-TC, 01115666-TA, 01115680, 01115681, 09171111, 09171135, 09171136 and 09171139, resulting tissue fragments were incubated in 3 ml Accumax (Innovative Cell Technologies, AM105) for 10 min at room temperature on a rocking shaker (ACC protocol). Cell suspensions were then filtered with a 100-μm cell strainer (Thermo Fisher Scientific, 08-771-19) and spun at 580*g* for 5 min at 4 °C. In cases where cell pellets appeared bloody, red blood cells were lysed with ACK Lysing Buffer (Thermo Fisher Scientific, A1049201) on ice for 1 min, followed by quenching with PBS and an additional centrifugation. The final cell pellet was resuspended in PBS (Thermo Fisher Scientific, MT21040CV) with 2% FBS (Gemini Bio-Products, 100-106).

For biopsies 01115655-TA, 01115665, 01115149-TC, 01115578-TA, 09171123, 09171144 and 09171146, tissue fragments were incubated in 2–3 ml Medium 199, Earle’s Salts (M199, Thermo Fisher Scientific, 11150059) with 1 mg ml^−1^ Collagenase 4 (Thermo Fisher Scientific, NC9836075) and 10–20 μg ml^−1^ DNase I (StemCell Technologies, 7900) for 5–10 min in a 37 °C water bath with intermittent mixing, followed by additional mixing and pipetting (CD protocol). Cell suspensions were then filtered with a 100-μm cell strainer, spun at 580*g* for 5 min at 4 °C and the resulting pellet resuspended in PBS with 2% FBS. The blood clot from biopsy 09171144 was processed in a similar manner, with the exception that red blood cells were lysed with ACK Lysing Buffer on ice at 5-min increments for a total of 15 min. For the bone marrow aspirate from biopsy 09171144, mechanical and enzymatic dissociation were not performed and red blood cells were lysed with ACK Lysing Buffer on ice at 5-min increments for a total of 10 min.

### Single-cell sorting

Dissociated single cells were enriched for cancer cells, CD45^+^ immune cells and, for 09171144, other populations. Single-cell suspensions in PBS with 2% FBS were stained by incubating for 15 min at room temperature protected from light with anti-human PTPRC (CD45) monoclonal antibody conjugated to FITC (1:200 dilution, VWR ABNOMAB12230), anti-human EPCAM antibody conjugated to PE (1:50 dilution, Miltenyi Biotec, 130-091-253) and either Calcein-AM (1:200 dilution, Thermo Fisher Scientific, C3100MP; biopsies 01115655-TA and 01115665), 7-aminoactinomycin D (7-AAD) (1:200 dilution, Thermo Fisher Scientific, A1310; all other biopsies except sample 01115149-TC) or both (sample 01115149-TC). We first sorted cells with biological dimensions (high FSC-A and high SSC-A), selected single cells and excluded doublets or triplets (low FSC-W). Next, we sorted live cells (low 7AAD/ high Calcein-AM) that were CD45^+^ (high FITC, enriching for immune cells), EPCAM^+^ (high PE, enriching for cancer cells) or double negative (low FITC/low PE, only in biopsy 09171144) (Supplementary Fig. 8 shows example gating). When possible, we collected at least one full plate of each gating, but as we did not collect all live cells, the representation of immune versus cancer cells in our dataset do not reflect composition within tumors. Cell sorting was performed using a BD Biosciences FACSAria cell sorter (IIu or UV) with FACSDiva software. Individual cells were sorted into the wells of 96-well plates with 10 μl TCL buffer (QIAGEN, 1070498) with 1% β-mercaptoethanol (Sigma, 63689) per well. Plates were then sealed, vortexed for 10 s, spun at 3,700 r.p.m. for 2 min at 4 °C and frozen on dry ice.

### Transcriptome sequencing, alignment and quantification

Library preparation for bulk RNA-seq was performed using the Illumina TruSeq Stranded mRNA Sample Preparation kit (except for biopsy 01115149-TA, which was prepared using the unstranded Illumina TruSeq RNA Sample Preparation protocol (Revision A, 2010)). Libraries were sequenced with 101 bp paired-end reads (except biopsy 01115149-TA, which was sequenced with 76 bp paired-end reads) on an Illumina instrument.

For scRNA-seq, RNA was captured from single-cell lysates with 2.2× RNAClean SPRI beads (Beckman Coulter Genomics) without the final elution. After air drying and secondary structure denaturation at 72 °C for 3 min, library construction was performed using a slightly customized Smart-seq2 protocol^12^ with 21 cycles of PCR for preamplification. Complementary DNA (cDNA) was purified with 0.8× Ampure SPRI beads (Beckman Coulter Genomics) and eluted in 21 μl TE buffer. During tagmentation and PCR amplification, we used 0.2 ng of cDNA per cell and one-eighth of the Illumina NexteraXT (Illumina FC-131-1096) reaction volume. Individual cells were sequenced to a mean depth of ~1.5 million 38 bp paired-end reads on an Illumina NextSeq 500 instrument with 75 cycle high output kits (Illumina TG-160-2005).

After adaptor trimming, reads were aligned using STAR aligner v.2.7.2b^45^ with parameters: --outFilterMultimapNmax 20 --outFilterMismatchNmax 999 --outFilterMismatchNoverReadLmax 0.04 --alignIntronMin 20 --alignMatesGapMax 1250000 --alignIntronMax 1250000 --chimSegmentMin 12 --chimJunctionOverhangMin 12 --alignSJstitchMismatchNmax 5 -1 5 5 --chimMultimapScoreRange 3 --chimScoreJunctionNonGTAG -4 --chimMultimapNmax 20 --chimNonchimScoreDropMin 10 --peOverlapNbasesMin 12 --peOverlapMMp 0.1 --chimOutJunctionFormat 1. sjdbOverhang was set to 1 less than the untrimmed read length. We used multi-sample two-pass mapping for all samples from each patient, first mapping all samples (bulk and single-cell transcriptomes), merging the SJ.out.tab files, then running the second pass with the jointly called splice junctions. For samples with germline and/or somatic variant information available from bulk exome sequencing, we supplied a VCF specifying single-nucleotide variants for STAR’s variant-aware mapping via the --varVCFfile parameter. STAR BAMs were passed into Salmon v.0.14.1 (ref. ^46^) to generate gene-level TPM quantifications with parameters: --incompatPrior 0.0 --seqBias --gcBias --reduceGCMemory --posBias. STAR chimeric junctions were supplied to STAR-Fusion^47^ v.1.7.0 in kickstart mode to call ETS family fusions.

### Single-cell quality control and clustering

After removing low quality cells (<500 or >10,000 detected genes, <50,000 reads or >25% expression from mitochondrial genes), we used Seurat v.3.1.0 (ref. ^48^) to perform first-pass clustering using the TPM matrix rescaled to exclude mitochondrial genes. We manually identified and removed a small number of cells with anomalous expression patterns (chiefly coexpression of high levels of hemoglobin with marker genes for nonerythroid cells). Additionally, some cells that did not cluster with erythroid cells (easily identified with dominant hemoglobin expression) nonetheless had low levels of hemoglobin detected, suggestive of contamination from ambient RNA released from lysed erythroid cells. To account for this, we identified genes whose expression was correlated (Pearson correlation > 0.2) with total hemoglobin expression levels in nonerythroid cells with detectable hemoglobin. This consisted of a small set of genes with known function in erythroid cell development and function: *AHSP*, *GATA1*, *CA1*, *EPB42*, *KLF1*, *SLC4A1*, *CA2*, *GYPA*, *TFR2*, *RHAG*, *FAXDC2*, *RHD*, *ALAS2*, *SPTA1* and *BLVRB*. To mitigate batch effects driven by different degrees of contaminating ambient erythroid transcripts, we removed these genes, along with the genes encoding hemoglobin subunits, from the expression matrix for all nonerythroid cells. Following this, we did not observe strong batch effects, as evidenced by co-mingling of nonmalignant cells from different biopsies (Extended Data Fig. 1d).

We repeated the clustering and conducted all downstream analyses with the filtered expression matrix. After joint clustering of all cells (Extended Data Fig. 1c), we performed subclustering on three cell subsets: (1) NK and T cells, (2) B-lineage cells and (3) myeloid cells. We manually labeled clusters by dominant cell identity, as assessed by marker gene expression patterns (Extended Data Fig. 2 and Supplementary Table 4). Briefly, cancer cell clusters (*n* = 836, from 12 biopsies) were identified by expression of *AR* and *KLK3* (adenocarcinoma markers) or *CHGA* (NEPC marker); T cell populations by *CD3D* and *CD3G*; regulatory T cells by *CD4*, *FOXP3* and *CTLA4*; NK cells by absence of *CD3D* and *CD3G* and expression of *FCGR3A*, *FCGR3B* and *GZMB*; erythroid cells by *HBA* and *HBB*; neutrophils by *ELANE*, *CEACAM8*, *AZU1* and *DEFA1*; macrophages by *APOE*, *C1QA* and *C1QB*; monocytes by *ITGAX*, *CD14*, *FCGR3A* and *FCGR3B*; B cells by *CD19* and *MS4A1*; plasmablasts by *CD19* and absence of *MS4A1*; and plasma cells by *SDC1* and high expression of immunoglobulin genes. Additionally, we confirmed the identity of cancer cell clusters by matching transcriptome-inferred copy number alteration profiles generated from inferCNV v.0.99.7 (https://github.com/broadinstitute/inferCNV) with those obtained from corresponding bulk whole exome sequencing.

### AR isoform-specific reads

Our scRNA-seq dataset had relatively even sequencing coverage along transcripts as is characteristic of Smart-seq2. To identify reads that uniquely map to an AR isoform, we generated a FASTA transcriptome annotation of spliced sequences from isoforms described in literature (Supplementary Fig. 1)^2,49–55^. This allowed us to use a mapping-based approach to quantify reads mapping to specific AR isoform transcripts. This approach is more comprehensive than approaches considering only junction reads and it yields estimates of AR-V7 abundance that are highly correlated with published estimates in existing cohorts (Supplementary Fig. 2a,b). We extracted all reads initially mapped by STAR (using the GENCODE transcriptome annotation) to the *AR* genomic interval (and flanking intergenic region) X:66753830-67011796 and then remapped them to our AR isoform transcriptome, disallowing clipping, multimapping or chimeric reads and requiring end-to-end mapping (STAR parameters: --outFilterMultimapNmax 1 --alignEndsType EndToEnd --alignSoftClipAtReferenceEnds No --outFilterMismatchNmax 999 --outFilterMismatchNoverReadLmax 0.04 --peOverlapNbasesMin 10 --scoreGap -1000000). As our AR isoform annotation corresponded to transcript sequences after splicing, we set scoreGap to prohibitively penalize gaps introducing additional inferred splice events. For patients with germline or somatic *AR* single-nucleotide variants detected either via whole exome sequencing or OncoPanel, we transformed single-nucleotide variants to transcript coordinates and supplied this as a VCF to STAR using the --varVCFfile parameter for STAR’s variant-aware mapping. We reported all reads that mapped uniquely to an isoform with at most one mismatch in Fig. 1a.

Alignment to specific isoforms requires detection of identifying transcript features (for example a unique exon–exon boundary, a portion of an exon not in other isoforms or a unique combination of exons). Illumina reads are not long enough to cover full transcripts in a single read, so not all isoforms are equivalently detectable. For example, reads cannot be attributed to AR-FL, as inclusion of all its component exons is impossible in Illumina reads and it has no unique splice junctions or exons. Thus, we avoid comparing frequencies of isoform-specific reads from different isoforms in the same sample/cell. We can, however, compare levels of the same isoform across samples/cells.

Validation in the three bulk cohorts proceeded in the same fashion, except that for the 4 healthy prostate samples, the initial STAR genomic alignment step was run with --twopassMode Basic. Reads were downloaded as FASTQs for healthy prostate (ArrayExpress accession E-MTAB-1733) and TCGA (ISB-CGC, https://isb-cgc.appspot.com/). CRAMs for the Abida et al. cohort^8^ were downloaded from Amazon S3 (dbGaP accession code, phs000915.v2.p2) and converted to FASTQs with samtools fastq. Survival analyses incorporating AR variant expression were performed using the Python package lifelines v.0.23.9.

To test for AR-45 and AR-V7 mutual exclusivity, we permuted assignments of *AR* region reads to single cells for all cells with any isoform-specific reads, while keeping fixed the *AR* read depth for each cell. This represents a null distribution under which all cells from a biopsy have identical *AR* isoform expression patterns while preserving the per-cell *AR* expression levels observed in our data. We performed 10,000 random permutations and compared the number of cells in which there was AR-45 and AR-V7 coexpression (at least one isoform-specific read for each) under the null to that observed in our data.

### Gene set scoring

For both bulk samples and single cells, we scored the activity of gene sets with VISION v.2.0.0 (ref. ^27^). Curated gene sets are shown in Supplementary Table 5. The hallmark gene sets were obtained from v.7.0 of the Molecular Signatures Database (MSigDB)^16^.

Analyzing cancer cell scRNA-seq data collected from patient biopsies poses statistical challenges. Cancer cells generally show patient-specific expression patterns that cause two cancer cells from a given patient to resemble each other more than any two cancer cells from different patients. This is driven substantially by patient-specific patterns of large somatic copy number alterations, which cause changes in expression of the affected genes to a degree that the copy number events can be inferred from scRNA-seq and used to cluster patient cancer cells. We aimed to uncover common, generalizable changes in patterns of expression related to enzalutamide exposure and sought to downweigh phenomena unique to individual patients. Patient-specific mechanisms can be detected with high power when cancer cells from a biopsy are particularly unusual or when they represent a larger than typical fraction of the total cohort of cells. To counter these effects, we developed a subsampling procedure detailed next and visualized in Supplementary Fig. 3. By considering subsets of the data more balanced for representation from different biopsies, we traded reduced power for more robustness.

When comparing VISION scores in cells from biopsies exposed and naive to treatment with enzalutamide, we included only cells inferred to be in G1 by Seurat to reduce discovery of signals introduced by different proportions of cycling cells between tumors^48^. We restricted our initial analyses to biopsies with at least ten G1 cancer cells. From either class (enzalutamide-naive versus exposed), we sampled up to 20 cells per biopsy to prevent results from being dominated by tumors with many recovered cells. Additionally, across repeated sampling iterations, we took a leave-one-out sampling procedure across biopsies. We omitted each biopsy in turn, instead sampling cells from other biopsies within its class, keeping the total number of cells the same. We performed 501 iterations of sampling for each biopsy being excluded. For each gene set being scored with VISION, we used the sampling with the median effect size as the summary of all iterations. When measuring effect size, we consistently compared one class versus the other (that is always exposed relative to naive) to ensure consistency in comparisons of direction of effect. We used the corresponding two-sided Mann–Whitney *U*-test *P* value as the nominal *P* value for the given gene set.

We additionally took the following steps to filter results that seemed to be driven by a single biopsy: for any given biopsy, we compared samplings when cells from the biopsy were held out versus when cells from the biopsy were included. If the proportion of nominally significant results (*P* < 0.05, same direction of effect as the overall median outcome for the given signature) when the biopsy was excluded was <80% of the proportion of nominally significant results when the biopsy was included, we considered any overall finding of differential gene set expression as nonrobust. This step was performed after computing Benjamini–Hochberg false discovery rate (FDR)^56^. Note that *P* values shown in Fig. 2b,c are based on all G1 cells and confirmed the findings from this sampling approach.

### Regulon activity

As only a single biopsy had a small cell carcinoma histology, we opted against gene-level differential expression analysis to avoid discoveries unique to one patient. Instead, we inferred transcriptional regulatory factor activity using all cancer cells from our cohort using SCENIC v.1.1.2.2^29^. This approach integrates information from patterns of gene expression covariation within our single-cell data with transcription factor binding site information to infer the sets of genes (regulons) upregulated by particular transcription factors and the activity of regulons within individual cells^29^. By focusing on inferred transcription factor activity as opposed to comparing individual genes, this approach has been previously shown to identify more generalizable patterns in expression programs in cancer^29^. In Fig. 3, for single cells, we used SCENIC AUC directly as a measure of regulon activity. For Fig. 3c,e, to infer regulon activity in bulk samples, we extracted gene sets corresponding to regulons from SCENIC and scored bulk samples for activity of the genes sets using VISION^8,18,27^.

We compared inferred regulon activities between small cell carcinoma and adenocarcinoma cells within our cohort using a similar subsampling approach as detailed previously for comparisons of gene set scores, except that in comparing SCENIC AUC scores, we did not restrict to only G1 cells, as the regulons had been inferred with all cancer cells together. As there was one small cell carcinoma biopsy, cells from that biopsy were never selected for omission across samplings.

### Signature scoring in bulk RNA-seq from Abida et al. cohort

Clinical annotations and expression quantifications were obtained from the published supplementary materials and from the authors directly^8^. We converted gene expression values from fragments per kilobase of transcript per million mapped reads (FPKM) (as supplied by the authors) to TPM for consistency with the rest of our study. As this cohort included samples sequenced at different centers and from different metastatic sites, we further restricted our analyses to avoid batch effects. For Fig. 2d, we analyzed only samples sequenced via transcriptome capture at the University of Michigan, as this was the largest identifiably uniformly sequenced subset. We could not analyze bone biopsies in this cohort due to paucity of post-enzalutamide samples. For Fig. 3c, as the largest number of small cell carcinoma samples were sequenced at Cornell, we included only small cell carcinoma and adenocarcinoma cases from Cornell in our analyses.

### Cell culture and western blots

The enzalutamide-resistant cell line VCaP-16 was maintained in DMEM with 10% FBS and 16 μM enzalutamide (SelleckChem, S1250), while parental VCaP-D was maintained in DMEM with 10% FBS. VCaP-16 and VCaP-D cells were plated into six-well plates in maintenance medium at a density of 50% and allowed to attach for 48 h. VCaP-16 cells are available from S.P.B. upon request. Cells in assay wells were then washed twice with PBS and switched into basal maintenance medium with 0.5% FBS for 48 h (VCaP-16 basal medium still contains 16 μM enzalutamide). Select samples were pretreated with the TGF-β receptor inhibitor SB-431542 (SelleckChem, S1067) for the last 24 of the 48 h. Following the 48-h incubation period, cells were treated for 2 h with fresh 0.5% FBS maintenance medium containing SB-431542, recombinant TGF-β-1 protein (R&D Systems, 240-B-002), or a combination of both. After 2 h, medium was aspirated from all samples and each well was rinsed briefly in PBS.

Cells were lysed in RIPA buffer with protease and phosphatase inhibitor cocktail (Thermo Fisher Scientific, P178440) on a vortex shaker for 30 mins at 4 °C. Lysates were spun at 16,000*g* for 15 mins at 4 °C and supernatant containing the soluble protein fraction was transferred to a new tube. Protein lysates were added to loading buffer (2× Laemmli Sample Buffer, Bio-Rad, 1610737) and the samples were boiled at 90 °C for 7 mins. Samples were electrophoresed at 200 V using a 4–20% denaturing PAGE gel (Bio-Rad). Relative protein levels of phospho-SMAD2 (Ser465/467), SMAD2/3 and vinculin were determined via immunoblotting using the following antibodies/dilutions: phospho-SMAD2 (Ser465/467) (138D4) antibody (Cell Signaling Technologies, 3108T) at 1:1,000 dilution, SMAD2/3 antibody (Cell Signaling Technologies, 3102) at 1:1,000 dilution and vinculin antibody (7F9) (Santa Cruz Biotechnology, sc-73614) at 1:10,000 dilution. The experiment and blotting was performed once.

To assess response to short-term enzalutamide exposure, wild-type VCaP cells were seeded in DMEM with 10% FBS in 24-well plates and allowed to attach for 24 h. The cells were treated with 10 μM enzalutamide for 24, 48 or 72 h or DMSO for 72 h. Recombinant TGF-β-1 protein was added 1 h before collecting cells for lysis and western blot. Antibodies are as previously stated, with the addition of anti-PSA antibody (rabbit polyclonal, Biodesign, K9211DR) at 1:10,000 dilution and β-actin HRP-conjugate antibody (Abcam, ab20272) at 1:20,000 dilution.

### pSMAD2 immunohistochemistry

Samples frozen in OCT were cryosectioned at 5-μm thickness, fixed in 10% neutral buffered formalin for 12 h and washed in 1× PBS. For each biopsy, slides were cut from multiple tissue blocks (*n* = 2–6, depending on available tissue) and evaluated for tumor content, with representative images shown from regions with sufficient tumor cellularity. Immunohistochemical staining of phospho-SMAD2 was performed on the automated Leica Bond RX system, using the Bond Polymer Refine kit with anti-phospho-SMAD2 (Ser465/467) antibody (E8F3R, rabbit monoclonal, Cell Signaling Technology) at a dilution of 1:100 overnight at 4 °C.

### HOXB5, HOXB6, NR1D2 immunohistochemistry

Patient-derived organoids of NEPC were developed from metastatic biopsies as previously described^57^. NEPC organoids (OWCM154, OWCM155, OWCM1078 and OWCM1262) and prostate adenocarcinoma (LNCaP and VCaP) xenograft models were formalin-fixed, paraffin embedded and serial sectioned at 4 μm thickness. Formalin-fixed, paraffin embedded slides were immersed in xylene and ethanol solutions for deparaffinization and dehydration. Antigens were retrieved in boiled 10 mM sodium citrate buffer (Sigma-Aldrich). Slides were blocked using horse serum and incubated with primary antibodies at 4 °C overnight. Slides were washed and developed using the ABC kit (Vector Laboratories), followed by hematoxylin counterstaining. Each stain was performed twice independently on the same batch of freshly sectioned slides and at least three images were taken from each in low and high magnification, with representative images shown in Fig. 3f. Dilutions for primary antibodies were anti-NR1D2 (1:50, NOVUS, NBP2-56141), anti-HOXB5 (1:10, Sigma, HPA043851) and anti-HOXB6 (1:200, Abcam, ab219499).

### TCR reconstruction

We performed TCR reconstruction and clonotype inference from scRNA-seq with TraCeR v.0.6.0 (ref. ^58^). We performed TCR reconstruction and estimation of clonal fraction from bulk RNA-seq using MiXCR v.3.0.12 (ref. ^59^). TCRs were inferred as detected in both bulk and scRNA-seq if the CDR3 nucleic acid (and therefore amino acid) sequence matched.

### Expression of prostate cancer risk genes

For Extended Data Fig. 5a, we examined expression of a published set of prostate cancer risk genes^38^. For each cluster of cells, we computed the fraction of cells expressing each gene at TPM ≥ 1. For nonmalignant cells, we combined cells from different biopsies in each cluster. For cancer cells, we considered each biopsy separately as its own cluster. We further grouped cell clusters into ‘superclusters’ of related cell types (Supplementary Table 4 and Extended Data Fig. 5). We deemed a gene to be cell type-specific if, in the cluster where it was most frequently expressed, it was expressed in at least a quarter of the cells and if the proportion expressing was at least 2.5-times that of every cluster in any other supercluster. This allows the identification of, for example, *PIK3C2B*, which is highly expressed in both B cells and plasmablasts but only sparsely in unrelated cell types.

### Cluster-specific genes and LDSC-SEG

LDSC-SEG considers genome-wide signals of disease association, including mostly weak, nonsignificant signals and determines whether they are significantly concentrated in subsets of the genome. More specifically, LDSC-SEG examines heritability in regions of the genome surrounding genes specifically expressed in different cell populations to nominate cell types relevant to disease biology^40^. We grouped cell clusters into ‘superclusters’ of related cell types as before and performed differential expression to identify markers for each cell cluster, omitting cells in the same supercluster. To mitigate uneven representation of cell types, when comparing against any cluster, we subsampled the same number of cells from each other supercluster and used as even a representation as possible of the contained clusters. In determining cancer cell markers, we used as even a representation as possible of cells from each biopsy while sampling 200 cancer cells total per iteration. For each cluster, we repeated sampling 500 times. In each sampling, we performed a one-sided Mann–Whitney *U*-test for differential expression on all genes with at least one TPM expression in at least 10% of the cluster’s cells. We then selected the top 10% most-upregulated genes (lowest median *P* value across samplings) as cluster specifically expressed genes. We used a 100 kb interval around genes for heritability partitioning with LDSC-SEG v.1.0.1, additionally including an annotation corresponding to all genes and the baseline v.1.1 model^40^.

### Reporting Summary

Further information on research design is available in the Nature Research Reporting Summary linked to this article.

## Online content

Any methods, additional references, Nature Research reporting summaries, source data, extended data, supplementary information, acknowledgements, peer review information; details of author contributions and competing interests; and statements of data and code availability are available at 10.1038/s41591-021-01244-6.

## Supplementary information

Supplementary Information Supplementary Figs. 1–8

Reporting Summary

Supplementary Tables Supplementary Tables 1–6

Supplementary Data scRNA-seq expression matrix (TPM).

## Data Availability

scRNA-seq expression and clustering data generated in this study are available at https://singlecell.broadinstitute.org/single_cell/study/SCP1244/transcriptional-mediators-of-treatment-resistance-in-lethal-prostate-cancer. Raw sequence data generated in this study are being deposited in dbGaP (accession phs001988.v1.p1). FASTQs for healthy prostate RNA-seq are from ArrayExpress under accession E-MTAB-1733. TCGA data are available at dbGaP accession phs000178.v11.p8. Abida et al. data are available at dbGaP accession phs000915.v2.p2. The hallmark gene sets from MSigDB can be accessed at https://www.gsea-msigdb.org/gsea/msigdb/collections.jsp#H. Source data are provided with this paper.

## Source data

Source Data Fig. 2 Unprocessed western blots for Fig. 2e.
