# Supplementary material for: Transcriptional mediators of treatment resistance in lethal prostate cancer
Source: Nat Med. 2021 Mar 4;27(3):426–33. doi: 10.1038/s41591-021-01244-6 (PMC7960507; doi:10.1038/s41591-021-01244-6)
Supplement: Supplementary file 1 — Supplementary Figs. 1–8 [file 41591_2021_1244_MOESM1_ESM.pdf]

nature portfolio

https://doi.org/10.1038/s41591-021-01244-6

Supplementary information

Transcriptional mediators of treatment resistance in lethal prostate cancer

In the format provided by the authors and unedited

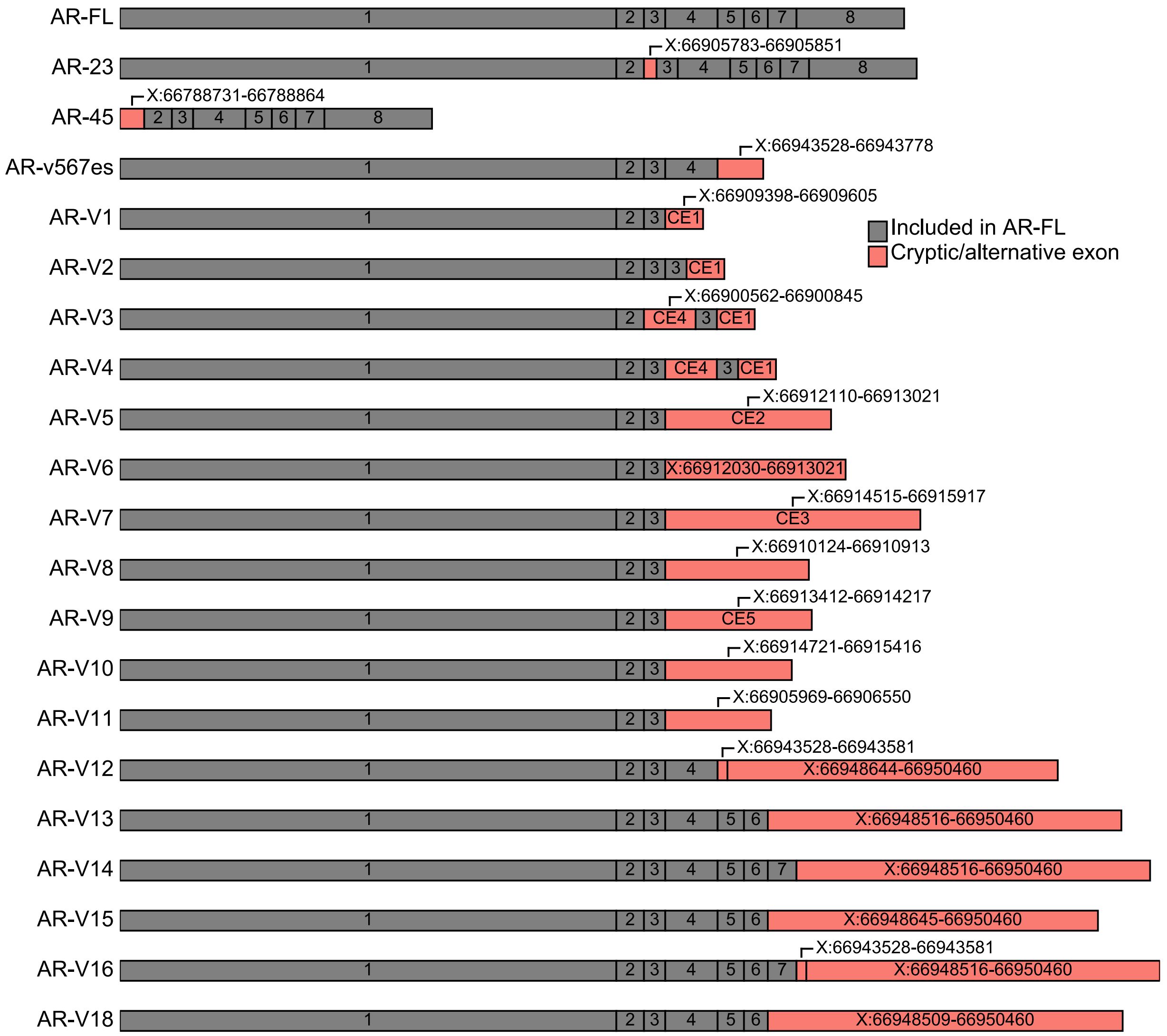

Supplementary Figure 1. Structure of AR splice variants curated from literature

Exons present in AR-FL are numbered. Alternative exons used in splice variants are labeled with corresponding genomic coordinates and by commonly used name if appearing more than once. Note that AR-V17 was omitted, as it was identified in a breast cancer cell line. For references, see Methods.

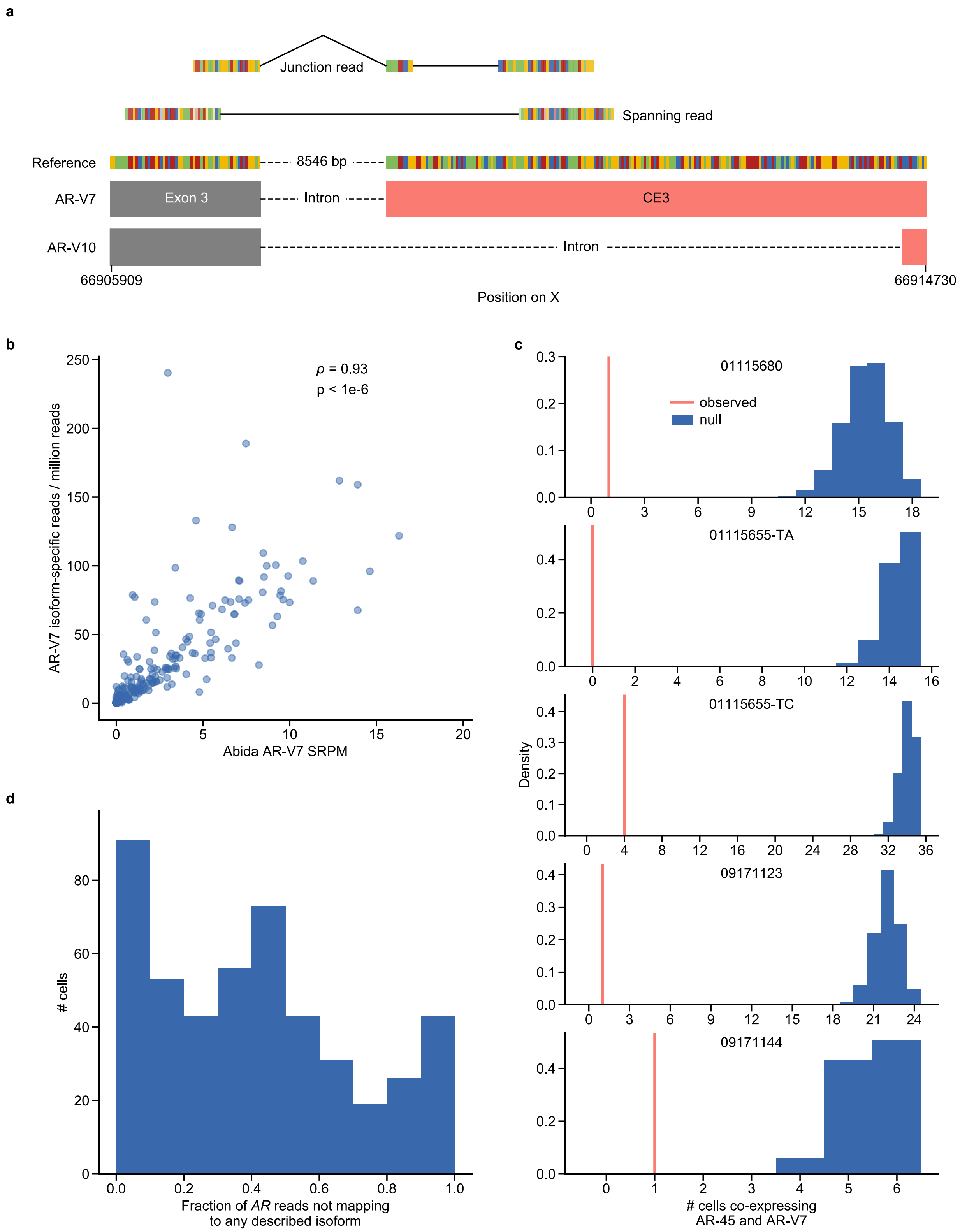
a
Junction read
Spanning read
Reference
8546 bp
AR-V7
Exon 3
Intron
CE3
AR-V10
Intron
66905909
66914730
Position on X
b
ρ = 0.93
p < 1e-6
AR-V7 isoform-specific reads / million reads
Abida AR-V7 SRPM
c
01115680
observed
null
01115655-TA
01115655-TC
Density
09171123
09171144
cells co-expressing
AR-45 and AR-V7
d
cells
Fraction of AR reads not mapping
to any described isoform

Supplementary Figure 2. AR isoform-specific mapping

a) Both junction reads that directly cover exon-exon boundaries and spanning reads that do not can be isoform-informative. Shown are two read pairs from our single-cell dataset that map specifically to AR-V7. Nearby exons from our curated *AR* transcriptome reference are shown.
b) AR-V7 isoform-specific read frequency derived using our approach is highly correlated to published AR-V7 splice reads per million (SRPM) metrics in a published bulk prostate bulk RNA-seq cohort[8]. ρ: Spearman's rank correlation coefficient; p value from computing Spearman's ρ on 1 million random permutations.
c) Observed numbers of single cells co-expressing AR-45 and AR-V7 (in biopsies where both are detected) is significantly lower than expected under null ($p < 1e\text{-}4$; Methods).
d) Proportion of reads that mapped to the *AR* genomic locus that did not map to any of the literature described isoforms in adenocarcinoma cells from our single-cell cohort.

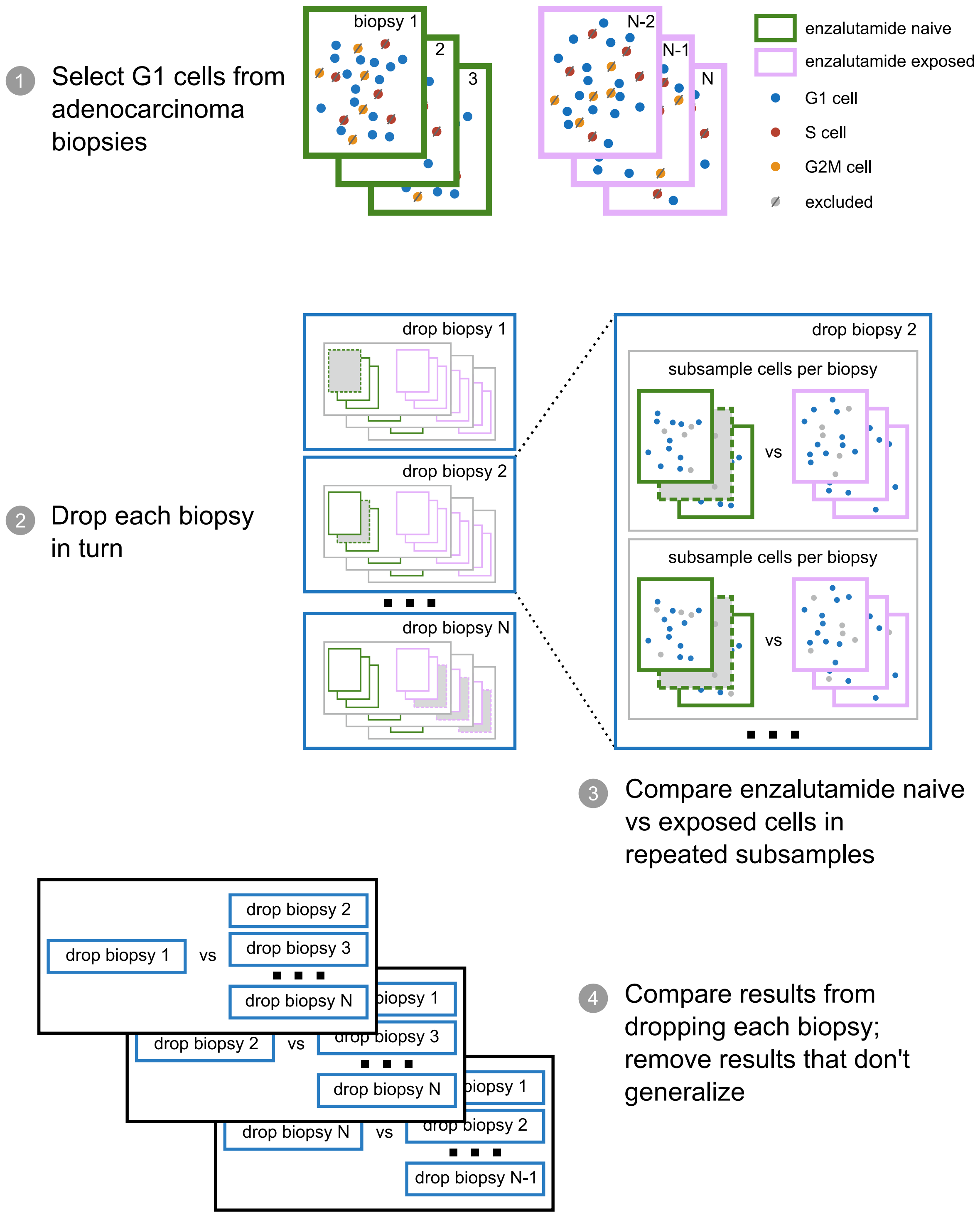

Supplementary Figure 3. Subsampling procedure to identify generalizable expression changes in cancer cells

See Methods § Gene set scoring for details.

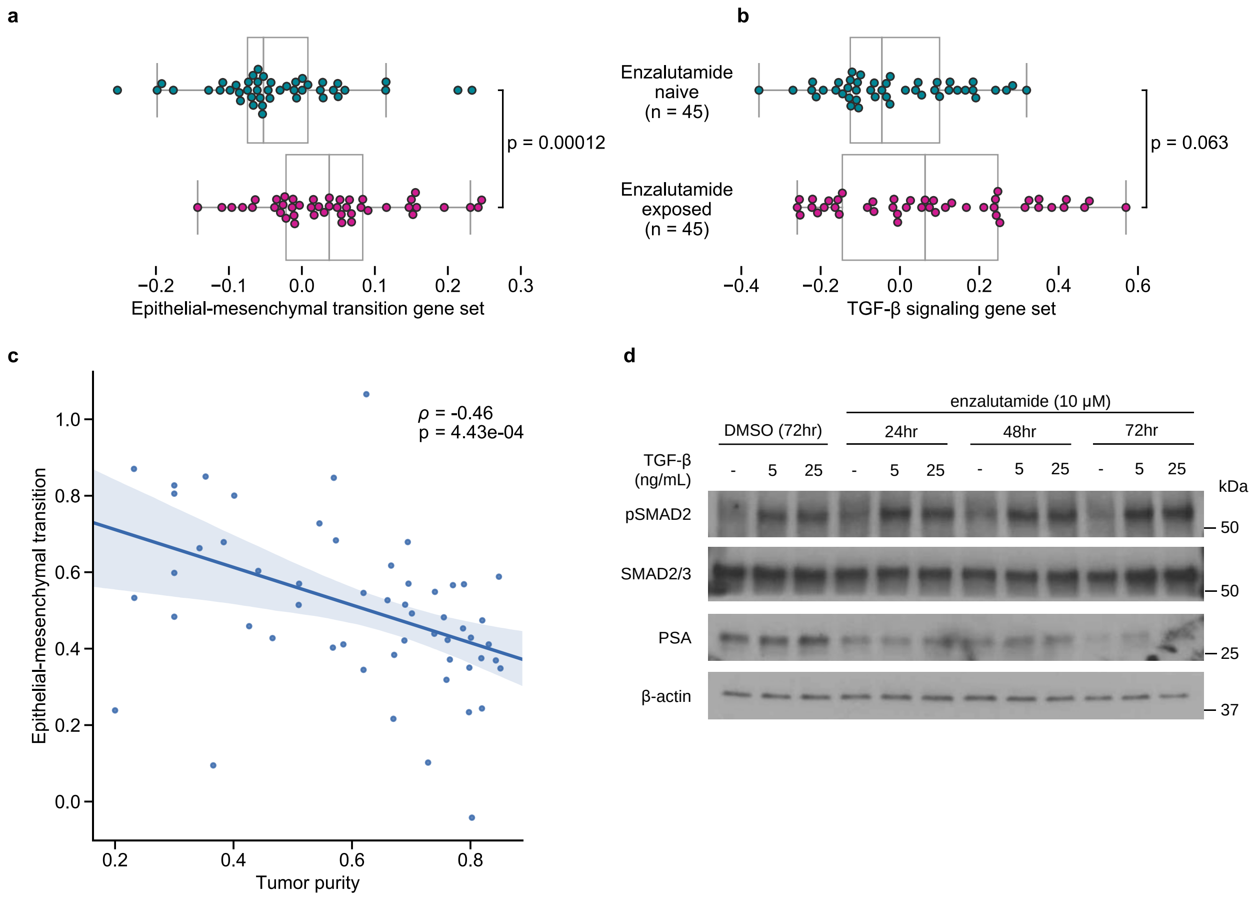

Supplementary Figure 4. Epithelial-mesenchymal transition and TGF-β signaling gene signature expression

Expression scores for MSigDB Hallmark **a)** epithelial-mesenchymal transition and **b)** TGF-β signaling gene sets from G1 cells collected before and after enzalutamide exposure from patient 01115655. These are a subset of the cells included in Fig. 2b, c. Boxplots: center line: median; box limits: upper and lower quartiles; whiskers extend at most 1.5x interquartile range past upper and lower quartiles. *p* values from two-sided Mann-Whitney *U* test.
c) In bulk samples, tumor purity is inversely correlated with MSigDB Hallmark epithelial-mesenchymal signature scores. Expression taken from a published cohort with tumor purity from an earlier publication with overlapping samples[8,28]. All displayed points are from lymph node metastases. Robust regression line is plotted with 95% confidence intervals estimated from a 5000-iteration bootstrap procedure. ρ: Spearman's rank correlation coefficient. *p* value represents fraction of 1 million random permutations with Spearman's ρ with absolute value greater than that of observed ρ.
d) Western blot of SMAD2/3, phospho-SMAD2, and PSA levels in wildtype VCaP cells after 24, 48, or 72 hours of enzalutamide exposure treated with TGF-β1. As expected, enzalutamide exposure decreases PSA protein levels. Western representative of 2 replications of experiment. For unprocessed images, see accompanying Source Data (pg 11).

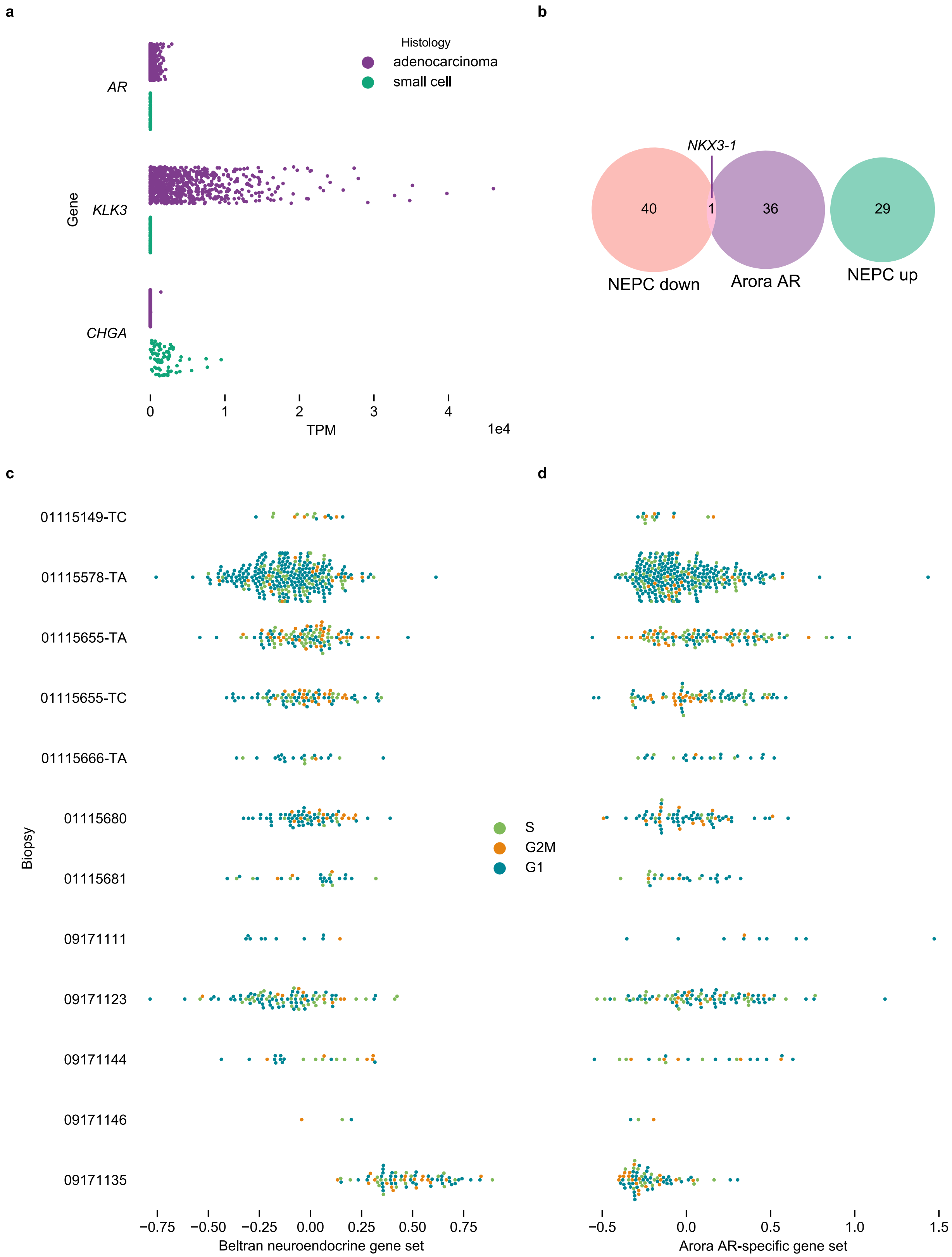
a
Histology
adenocarcinoma
small cell
AR
KLK3
CHGA
Gene
0
1
2
3
4
TPM
1e4
b
NKX3-1
40
1
36
29
NEPC down
Arora AR
NEPC up
c
d
01115149-TC
01115578-TA
01115655-TA
01115655-TC
01115666-TA
01115680
01115681
09171111
09171123
09171144
09171146
09171135
Biopsy
S
G2M
G1
−0.75
−0.50
−0.25
0.00
0.25
0.50
0.75
Beltran neuroendocrine gene set
−0.5
0.0
0.5
1.0
1.5
Arora AR-specific gene set

Supplementary Figure 5. Adenocarcinoma and small cell carcinoma cells have diverging marker gene and gene signature expression.

a) *AR* and *KLK3* (which encodes PSA) expression marks adenocarcinoma cells (n = 760), while *CHGA* marks small cell carcinoma cells (n = 76).
b) Number of genes in Arora AR-specific and Beltran neuroendocrine gene sets (Supplementary Table 5)[17,18]. The latter is a directional gene signature, including both genes up and down in NEPC relative to adenocarcinoma. There is only one shared gene between the Arora AR-specific and Beltran NEPC down sets: *NKX3-1*, a common marker of prostate adenocarcinoma.
Expression of **c)** Beltran neuroendocrine gene set and **d)** Arora AR-specific gene set by biopsy. Cancer cells from all cell cycle phases are included and scored for expression. 09171135 has a small cell carcinoma histology, while the others are adenocarcinomas.

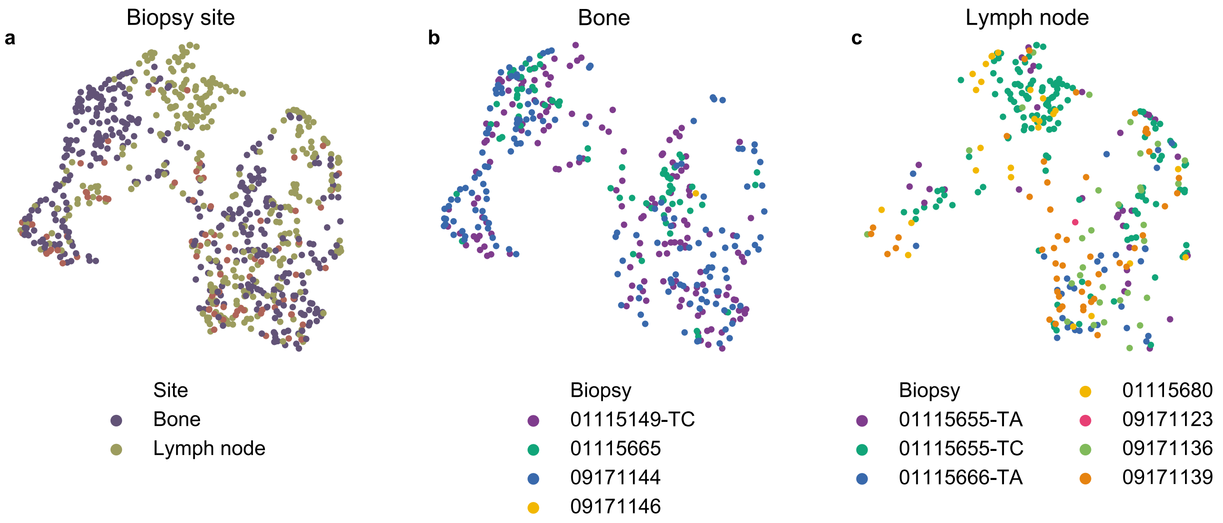

Supplementary Figure 6. Cytotoxic cells from across metastatic sites

NK and T cells are projected onto UMAP space as in Fig. 4a.
a) Cells are labelled by site of biopsy. Cells infiltrating **b)** bone and **c)** lymph node metastases are labelled by originating biopsy.

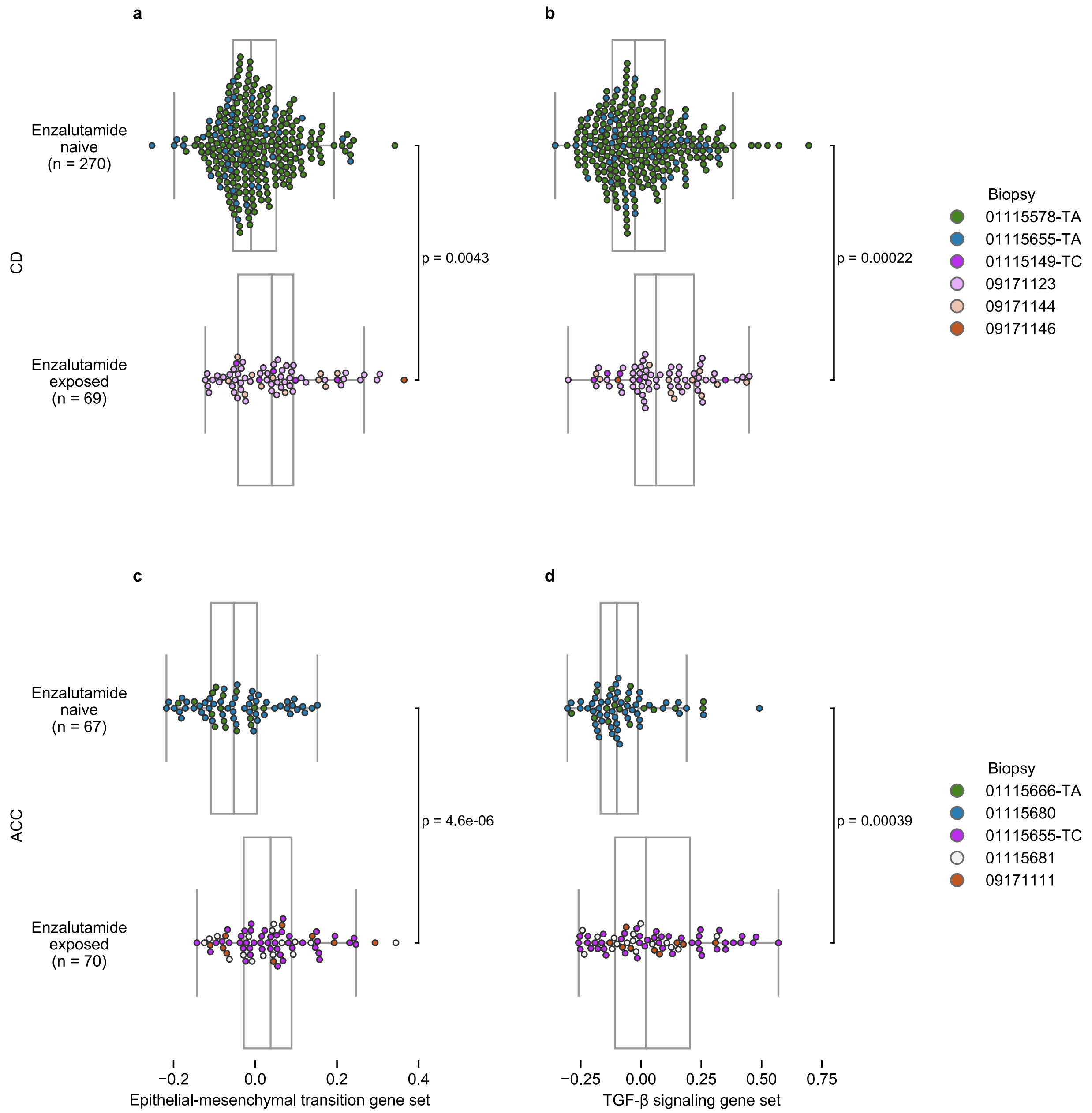

Supplementary Figure 7. Changes in expression programs after enzalutamide exposure are concordant across dissociation conditions.

a) Single-cell signature scores for MSigDB Hallmark epithelial-mesenchymal and **b)** TGF-β signaling gene sets for biopsies dissociated with the CD protocol and **c, d)** with the ACC protocol (Methods). Boxplots: center line: median; box limits: upper and lower quartiles; whiskers extend at most 1.5x interquartile range past upper and lower quartiles. p values from two-sided Mann-Whitney U test.

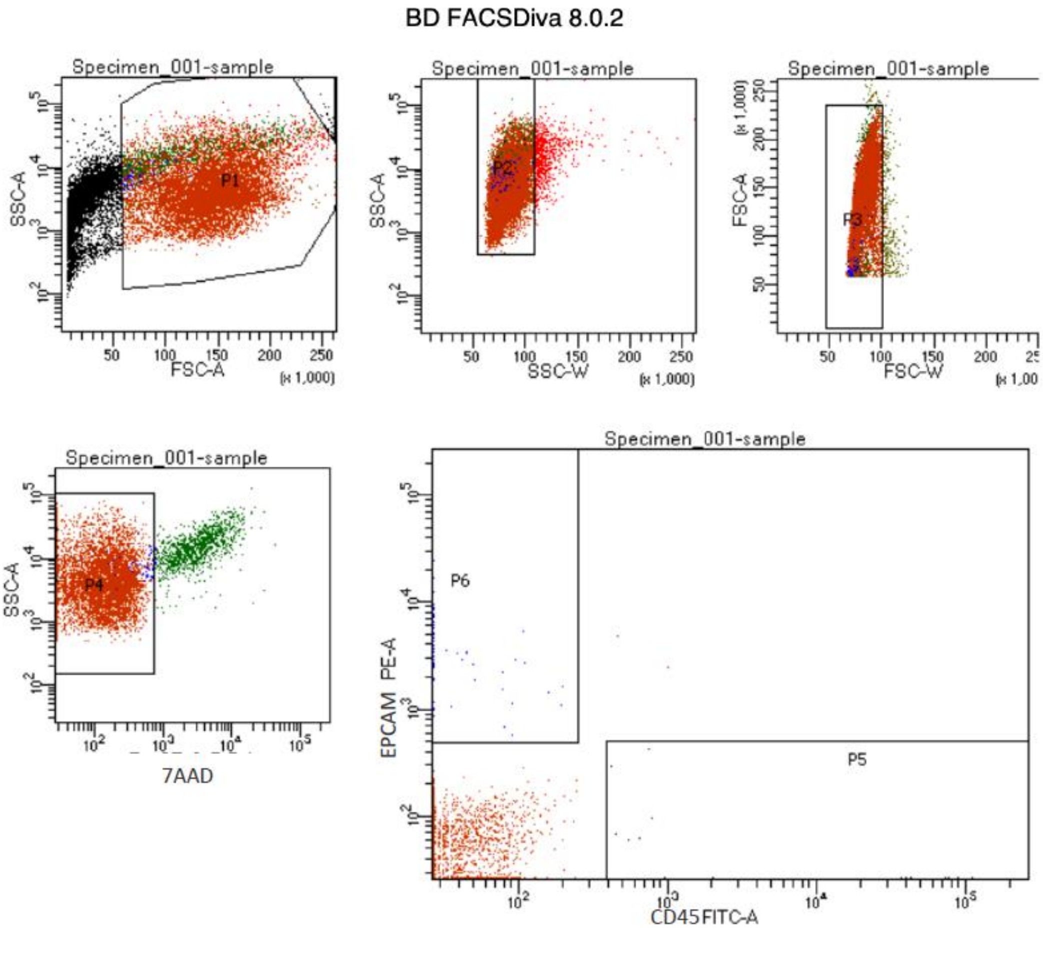

Tube: sample

Population	#Events	%Parent	%Total
All Events	20,731	####	100.0
P1	11,573	55.8	55.8
P2	10,844	93.7	52.3
P3	10,548	97.3	50.9
P4	9,432	89.4	45.5
P5	15	0.2	0.1
P6	61	0.6	0.3

Supplementary Figure 8. Example FACS gating strategy

Gating for biopsy 09171136 shown. See Methods for details.

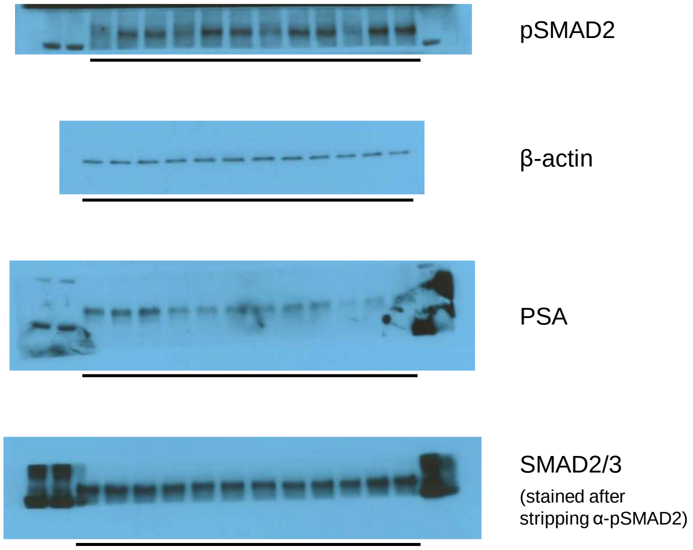

Source Data for Supplementary Fig. 4d
