# Supplementary material for: Transcriptional mediators of treatment resistance in lethal prostate cancer
Source: Nat Med. 2021 Mar 4;27(3):426–33. doi: 10.1038/s41591-021-01244-6 (PMC7960507; doi:10.1038/s41591-021-01244-6)
Supplement: Supplementary file 2 — Reporting Summary [file 41591_2021_1244_MOESM2_ESM.pdf]

nature research

Corresponding author(s): Eliezer M. Van Allen

Last updated by author(s): Jan 7, 2021

Reporting Summary

Nature Research wishes to improve the reproducibility of the work that we publish. This form provides structure for consistency and transparency in reporting. For further information on Nature Research policies, see Authors & Referees and the Editorial Policy Checklist.

Statistics

For all statistical analyses, confirm that the following items are present in the figure legend, table legend, main text, or Methods section.

n/a	Confirmed	
☐	☒	The exact sample size (*n*) for each experimental group/condition, given as a discrete number and unit of measurement
☒	☐	A statement on whether measurements were taken from distinct samples or whether the same sample was measured repeatedly
☐	☒	The statistical test(s) used AND whether they are one- or two-sided<br>*Only common tests should be described solely by name; describe more complex techniques in the Methods section.*
☒	☐	A description of all covariates tested
☐	☒	A description of any assumptions or corrections, such as tests of normality and adjustment for multiple comparisons
☐	☒	A full description of the statistical parameters including central tendency (e.g. means) or other basic estimates (e.g. regression coefficient) AND variation (e.g. standard deviation) or associated estimates of uncertainty (e.g. confidence intervals)
☐	☒	For null hypothesis testing, the test statistic (e.g. *F*, *t*, *r*) with confidence intervals, effect sizes, degrees of freedom and *P* value noted<br>*Give P values as exact values whenever suitable.*
☒	☐	For Bayesian analysis, information on the choice of priors and Markov chain Monte Carlo settings
☒	☐	For hierarchical and complex designs, identification of the appropriate level for tests and full reporting of outcomes
☒	☐	Estimates of effect sizes (e.g. Cohen's *d*, Pearson's *r*), indicating how they were calculated

Our web collection on statistics for biologists contains articles on many of the points above.

Software and code

Policy information about availability of computer code

Data collection

FACSDiva 8.0.2

Data analysis

General statistical computing & visualization:
SciPy v1.3.2
Python 3.7
Matplotlib v3.1.1
seaborn v0.9.0
R v3.5.1

Whole exome analyses:
BWA v0.5.9
Getz Lab CGA WES Characterization pipeline (https://portal.firecloud.org/#methods/getzlab/CGA_WES_Characterization_Pipeline_v0.1_Dec2018/2), which includes ContEst, MuTect v1.1.6, Strelka v1.0.11, DeTiN v1.8.5, Orientation Bias Filter v17, MAFPoNFilter, and Oncotator v1.9.9.0
DeepVariant v0.8.0
vcf2maf v1.6.17
FACETS v0.5.14

RNA analyses:
cutadapt v2.2
STAR v2.7.2b
Salmon v0.14.1
STAR-Fusion v1.7.0
inferCNV v0.99.7
VISION v2.0.0

SCENIC v1.1.2.2
TraCeR v0.6.0
MiXCR v3.0.12

Other:
Seurat v3.1.0
lifelines v0.23.9
LDSC-SEG v1.0.1

For manuscripts utilizing custom algorithms or software that are central to the research but not yet described in published literature, software must be made available to editors/reviewers. We strongly encourage code deposition in a community repository (e.g. GitHub). See the Nature Research guidelines for submitting code & software for further information.

Data

Policy information about availability of data

All manuscripts must include a data availability statement. This statement should provide the following information, where applicable:

- Accession codes, unique identifiers, or web links for publicly available datasets
- A list of figures that have associated raw data
- A description of any restrictions on data availability

scRNA-seq expression and clustering data generated in this study are available at https://singlecell.broadinstitute.org/single_cell/study/SCP1244/transcriptional-mediators-of-treatment-resistance-in-lethal-prostate-cancer. Raw sequence data generated in this study are being deposited in dbGaP (accession phs001988.v1.p1). FASTQs for healthy prostate RNA-seq are from ArrayExpress accession E-MTAB-1733. TCGA data are available at dbGaP accession phs000178.v11.p8. Abida et al. data are available at dbGaP accession phs000915.v2.p2. The Hallmark gene sets from the Molecular Signatures Database (MSigDB) can be accessed at https://www.gsea-msigdb.org/gsea/msigdb/collections.jsp#H.

Field-specific reporting

Please select the one below that is the best fit for your research. If you are not sure, read the appropriate sections before making your selection.

☒ Life sciences ☐ Behavioural & social sciences ☐ Ecological, evolutionary & environmental sciences

For a reference copy of the document with all sections, see nature.com/documents/nr-reporting-summary-flat.pdf

Life sciences study design

All studies must disclose on these points even when the disclosure is negative.

Sample size	2,170 post-QC cells from 14 patients and 15 biopsies. No sample-size calculation was performed a priori, as this is a descriptive study. We accrued biopsies from all available patients who consented to the study, and patient numbers are consistent with cancer scRNA-seq studies in literature.
Data exclusions	To arrive at 2,170 post-QC cells, sequenced cells were excluded from downstream analyses based on gene count (<500 or >10,000), read depth (< 50,000 reads), and expression patterns suggestive of ambient RNA contamination (see Methods for details). QC metric thresholds were not fixed before data analysis, but the QC procedure is consistent with current practices in field.
Replication	scRNA-seq data to replicate our findings are not directly available. While the comparisons are limited due to the mismatch in data type, we attempted to identify patterns of expression differences identified in our scRNA-seq data in bulk RNA-seq cohorts. Additionally, we used cell lines, patient biopsy tissues, and organoid models to test expression programs identified from sequencing analyses. VCaP-16 and VCaP-D TGF-β stimulation was tested at multiple concentrations and showed a clear dose-response relationship in both. pSMAD2 IHC was performed in pairs of pre/post-enzalutamide tissues from two separate patients. For each biopsy, slides were cut from multiple tissue blocks (n = 2-6, depending on available tissue) and evaluated for tumor content, with representative images shown from regions with sufficient tumor cellularity. HOXB5, HOXB6, NR1D2 immunohistochemistry was performed twice independently on the same batch of fresh sectioned slides and at least three images were taken from each in low and high magnification.
Randomization	This study did not involve allocation of patients/samples to interventions/experimental perturbations, so randomization did not apply.
Blinding	This study did not include experimental perturbations of biopsies. Comparisons of nuclear pSMAD2 staining in Fig. 2f were performed by a pathologist who was blinded to the timepoint label for each biopsy. There was no additional blinding, as this study did not involve prospective allocation of patients into arms/groups.

Reporting for specific materials, systems and methods

We require information from authors about some types of materials, experimental systems and methods used in many studies. Here, indicate whether each material, system or method listed is relevant to your study. If you are not sure if a list item applies to your research, read the appropriate section before selecting a response.

Materials & experimental systems

n/a	Involved in the study
☐	☒ Antibodies
☐	☒ Eukaryotic cell lines
☒	☐ Palaeontology
☒	☐ Animals and other organisms
☐	☒ Human research participants
☒	☐ Clinical data

Methods

n/a	Involved in the study
☒	☐ ChIP-seq
☐	☒ Flow cytometry
☒	☐ MRI-based neuroimaging

Antibodies

Antibodies used

anti-human PTPRC (CD45) monoclonal antibody conjugated to FITC (VWR #ABNOMAB12230)
anti-human EPCAM antibody conjugated to PE (Miltenyi Biotec #130-091-253)
anti-phospho-SMAD2 (Ser465/467) (138D4) antibody (Cell Signaling Technologies #3108T)
anti-SMAD2/3 antibody (Cell Signaling Technologies #3102)
anti-vinculin antibody (7F9) (Santa Cruz Biotechnology, #sc-73614)
anti-β-actin HRP-conjugate antibody (Abcam #ab20272)
anti-PSA antibody (rabbit polyclonal, Biodesign, Kennebunk, ME, #K9211DR)
anti-phospho-SMAD2 (Ser465/467) antibody (E8F3R, rabbit monoclonal, Cell Signaling Technology #18338)
anti-NR1D2 (NOVUS #NBP2-56141)
anti-HOXB5 (Sigma #HPA043851)
anti-HOXB6 (Abcam #ab219499)

Validation

For FACS, anti-human PTPRC (CD45) and anti-human EPCAM antibodies were validated by the manufacturers as specific for human and for use in flow cytometry.
Per manufacturer, anti-phospho-SMAD2 (Ser465/467) (138D4) detects endogenous levels of Smad2 only when dually phosphorylated at serines 465 and 467, and may detect Smad3 phosphorylated at the equivalent sites. This antibody does not cross-react with other Smad-related proteins. Species reactivity: human, mouse, rat, and mink. Validated for use in Western blot.
Per manufacturer, anti-SMAD2/3 antibody is recommended for use in Western blot and immunoprecipitation, with reactivity to human, mouse, rat, and monkey total Smad2/3 protein.
Per manufacturer, anti-vinculin antibody is recommended for use in Western blot, immunofluorescence, immunoprecipitation, and immunohistochemistry, with reactivity to vinculin of human, mouse, rat and avian origin.
Per manufacturer, anti-β-actin antibody is validated for use in Western blot and has specificity to mouse, rat, rabbit, chicken, cow, dog, human, pig, Drosophila melanogaster, African green monkey, and Chinese hamster.
anti-PSA antibody: specificity and sensitivity is supported by repeated results showing the detection of a protein band at the expected size. Protein detection was increased by androgen stimulation in prostate cancer cells and reduced by androgen receptor downregulation with siRNA or degrader or AR antagonist (e.g. enzalutamide).
anti-phospho-SMAD2 (Ser465/467) antibody (E8F3R): per manufacturer, antibody has specificity to human, mouse, and rat and is suitable for use in Western blot, immunoprecipitation, immunocytochemistry, flow cytometry, and ChIP. We performed titration experiments using 22Rv1 cells with and without TGF-β stimulation to determine antibody concentration.
anti-NR1D2, anti-HOXB5, anti-HOXB6: Antibodies for immunohistochemistry experiments were validated by the Human Protein Atlas (HPA) project according to manufacturer's website. We confirmed by serial dilution to determine specificity and expression location using manufacturer datasheet as reference. In addition, these antibodies are specific to human antigens but not mouse. We validated in patient derived xenografts showing no expression in mouse tissues.

Eukaryotic cell lines

Policy information about cell lines

Cell line source(s)

VCaP-16 cells were derived from long term culture of VCaP cells in enzalutamide (see Methods). VCaP cells were purchased from ATCC.

Authentication

VCaP cells were authenticated via DNA typing.

Mycoplasma contamination

VCaP cells tested negative for Mycoplasma via a DNA-based PCR test.

Commonly misidentified lines
(See ICLAC register)

None

Human research participants

Policy information about studies involving human research participants

Population characteristics

Patients were men who had metastatic castration resistant prostate cancer. No genomic features were used for participant selection. 13/14 patients had prostate adenocarcinoma, and 1/14 had small cell carcinoma. Biopsies were collected from metastases to bone (8/18), lymph node (9/18), and liver (1/18). All patients had received androgen deprivation therapy. Patients ranged in age from 57-79.

Recruitment	Patients were recruited as part of the research program at Dana-Farber Cancer Institute. The consenting patient population at this site may not necessarily be representative (e.g. potentially more urban) of all advanced prostate cancer patients; however, the genomic and clinical characteristics of our cohort are consistent with those of participants from large-scale bulk sequencing studies of mCRPC conducted in the US.
Ethics oversight	This study was approved by the Dana-Farber/Harvard Cancer Center Institutional Review Board under protocols # 09-171, 11-104, 13-301, and 01-045.

Note that full information on the approval of the study protocol must also be provided in the manuscript.

Flow Cytometry

Plots

Confirm that:

☒ The axis labels state the marker and fluorochrome used (e.g. CD4-FITC).

☒ The axis scales are clearly visible. Include numbers along axes only for bottom left plot of group (a 'group' is an analysis of identical markers).

☒ All plots are contour plots with outliers or pseudocolor plots.

☒ A numerical value for number of cells or percentage (with statistics) is provided.

Methodology

Sample preparation	Tumor samples were collected and transported in Dulbecco's Modified Eagle Medium, on ice. Single-cell suspensions for single-cell RNA-seq were obtained from tumor core needle biopsies through mechanical and enzymatic dissociation. Samples were first cut into pieces smaller than 1 mm3 using a scalpel. For bone biopsies, soft tissue was also scraped from the hard bone surface using a scalpel blade. Samples were then dissociated using one of two protocols, chiefly to optimize for yield of viable cells from different metastatic sites (Methods). Single cell suspensions in PBS with 2% FBS were stained by incubating for 15 minutes at room temperature protected from light with anti-human PTPRC (CD45) monoclonal antibody conjugated to FITC (1:200 dilution), anti-human EPCAM antibody conjugated to PE (1:50 dilution), and either Calcein-AM (1:200 dilution), 7-Aminoactinomycin D (7-AAD) (1:200 dilution), or both.
Instrument	BD Biosciences FACSAria cell sorter (IIu or UV)
Software	FACSDiva
Cell population abundance	Post-sort fractions included 45.5% viable cells (low 7AAD), 0.1% immune cells (high CD45-FITC), and 0.3% epithelial/tumor cells (high EPCAM-PE) as demonstrated by sample 09171136.
Gating strategy	We first sorted cells with biological dimensions (high FSC-A and high SSC-A), selected single cells, and excluded doublets or triplets (low FSC-W). Next, we selected live cells (low 7AAD/ high Calcein-AM) that were CD45+ (high FITC, enriching for immune cells), EPCAM+ (high PE, enriching for tumour cells), or double negative (low FITC/low PE).

☒ Tick this box to confirm that a figure exemplifying the gating strategy is provided in the Supplementary Information.
