# Supplementary material for: Transcriptional mediators of treatment resistance in lethal prostate cancer
Source: Nat Med. 2021 Mar 4;27(3):426–33. doi: 10.1038/s41591-021-01244-6 (PMC7960507; doi:10.1038/s41591-021-01244-6)
Supplement: Source Data Fig. 2 — Unprocessed western blots for Fig. 2e. [file 41591_2021_1244_MOESM5_ESM.pdf]

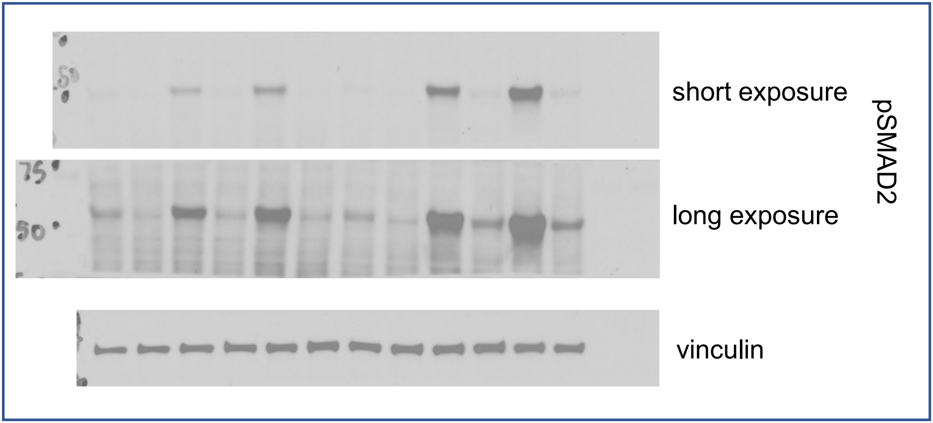

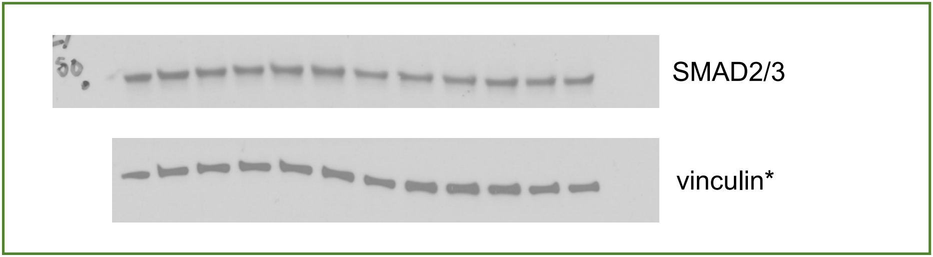

*pSMAD2 and SMAD2/3 have similar molecular weight and were stained using two different gels. This set of vinculin loading controls is not shown in Fig. 2e.
